# Multi-Degree Reduction of Said–Ball Curves and Engineering Design Using Multi-Strategy Enhanced Coati Optimization Algorithm

**DOI:** 10.3390/biomimetics10070416

**Published:** 2025-06-26

**Authors:** Feng Zou, Xia Wang, Weilin Zhang, Qingshui Shi, Huogen Yang

**Affiliations:** 1 School of Science, Jiangxi University of Science and Technology, Ganzhou 341000, China; 6120231206@mail.jxust.edu.cn (F.Z.); 6120231204@mail.jxust.edu.cn (X.W.); 6720231241@mail.jxust.edu.cn (W.Z.); 6120240981@mail.jxust.edu.cn (Q.S.); 2 Jiangxi Provincial Key Laboratory of Multidimension Intelligent Perception and Control, Jiangxi University of Science and Technology, Ganzhou 341000, China

**Keywords:** said-ball curve, degree reduction approximation, coati optimization algorithm, hybrid oppositional learning with good point set, fitness-distance balance, engineering design

## Abstract

Within computer-aided geometric design (CAGD), Said–Ball curves are primarily adopted in domains such as 3D object skeleton modeling, vascular structure repair, and path planning, owing to their flexible geometric properties. Techniques for curve degree reduction seek to reduce computational and storage demands while striving to maintain the essential geometric attributes of the original curve. This study presents a novel degree reduction model leveraging Euclidean distance and curvature data, markedly improving the preservation of geometric features throughout the reduction process. To enhance performance further, we propose a multi-strategy enhanced coati optimization algorithm (MSECOA). This algorithm utilizes a good point set combined with opposition-based learning to refine the initial population distribution, employs a fitness–distance equilibrium approach alongside a dynamic spiral search strategy to harmonize global exploration with local exploitation, and integrates an adaptive differential evolution mechanism to boost convergence rates and robustness. Experimental results demonstrate that the MSECOA outperforms nine highly cited agorithms in terms of convergence performance, solution accuracy, and stability. The algorithm exhibits superior behavior on the IEEE CEC2017 and CEC2022 benchmark functions and demonstrates strong practical utility across four engineering optimization problems with constraints. When applied to multi-degree reduction approximation of Said–Ball curves, the algorithm’s effectiveness is substantiated through four reduction cases, highlighting its superior precision and computational efficiency, thus providing a highly effective and accurate solution for complex curve degree reduction tasks.

## 1. Introduction

In the fields of computer-aided geometric design (CAGD) and computer graphics, significant progress has been made in shape optimization methods for parameterized free-form curves. The Ball curve, as one of the classic curves in CAGD, is primarily applied in mechanical design, vascular structure repair, and path planning due to its excellent properties in geometric modeling [1]. Since Ball introduced the rational cubic Ball curve in 1974 [2], research on Ball curves has continued to deepen. Wang extended the cubic Ball curve to higher-order generalized Ball curves [3], while Said further proposed the generalized Ball curve, extending it to arbitrary odd degrees and revealing its numerous advantageous properties in geometric modeling [4]. Hu et al. conducted an in-depth comparative study of generalized Ball curves and Bézier curves in terms of recursive evaluation and envelope properties [5]. In 2000, Wu introduced two new generalized Ball curves, namely the Said–Bézier generalized Ball curve and the Wang–Said generalized Ball curve (WSGB) [6]. Recently, Liu et al. proposed the h–Said–Ball basis function, enhancing modeling capabilities and improving evaluation efficiency through h-calculus [7]. These new curves exhibit more efficient recursive algorithms compared to traditional Bézier curves.

In curve approximation techniques, Sederberg and Farouki first introduced interval Bézier curves into curve approximation and formally proposed interval algorithms to ensure the stability and accuracy of computational results [8]. Tuohy et al. introduced the concept of interval B-spline curves and surfaces and studied the boundary properties of interval Bézier curves [8]. Lin and Rokne proposed disk Bézier curves and spherical Bézier curves, which demonstrated superior performance in geometric computations [9]. Lu et al. developed a Bézier curve degree reduction algorithm based on L2 norm approximation error control, significantly improving the stability and accuracy of degree reduction [10]. Quan et al. proposed a preprocessing asymptotic iterative approximation method to accelerate the convergence speed of tensor product Said–Ball surfaces, markedly enhancing computational efficiency and stability [11].

The degree reduction of curves and surfaces has long been a research focus in CAGD, particularly in complex geometric design, where the need for degree reduction is more pressing. Hu et al. conducted an in-depth study on the approximate degree reduction of Said–Ball curves and surfaces, proposing a stepwise degree reduction method, though it can only achieve single-step reduction at a time [5]. Chen and Lou developed a degree reduction algorithm for interval B-spline curves [12], while Tan and Fang systematically explored the degree reduction of interval WSGB curves [13]. Hu and Wang, based on Jacobi polynomials and quadratic programming theory, proposed an optimal multi-degree reduction algorithm [14]. Wang Hongkai achieved the exact degree reduction of Bézier curves through polynomial reparameterization, significantly simplifying the process and improving computational efficiency [15]. However, traditional degree reduction methods (e.g., least squares, geometric approximation, and classical optimization algorithms) exhibit significant limitations when handling complex curves like Said–Ball curves: first, their convergence speed is slow, failing to meet the demands of efficient computation; second, their accuracy is insufficient, particularly in high-dimension spaces where they tend to fall into local optima, making it difficult to simultaneously preserve geometric features and minimize errors; and third, their robustness is poor, lacking stability for complex shapes or high-degree curves, resulting in degree reduction outcomes that deviate from expectations. Moreover, traditional methods struggle to find globally optimal solutions when dealing with curves exhibiting multimodal or nonlinear characteristics, further limiting their applicability.

In view of the aforementioned challenges, the degree reduction problem can essentially be formulated as an optimization task, making it well-suited for resolution through bio-inspired intelligent optimization algorithms. In recent years, such algorithms have demonstrated significant potential in addressing complex optimization problems, particularly excelling in global search capabilities, convergence performance, and algorithmic robustness. These methods draw inspiration from natural phenomena, animal behaviors, and evolutionary mechanisms, abstracting biological principles into mathematical models to simulate adaptive and efficient search strategies. According to the No Free Lunch (NFL) theorem, no single algorithm can achieve optimal performance across all types of optimization problems [16]. This highlights the necessity of designing specialized algorithms tailored to specific application domains. To address this need, researchers have continuously proposed and refined bio-inspired intelligent optimization algorithms to deliver more efficient and targeted solutions. For instance, Cheng et al., based on uniform experimental design theory, proposed an improved sparrow search algorithm (ISSA), inspired by the foraging behavior of sparrows. By incorporating a surrounding diversity metric, dynamic management strategies, and boundary update mechanisms, ISSA enhances population diversity and convergence efficiency and has been validated for its superior performance in UAV path planning [17]. Mahmoud et al. developed an enhanced hiking optimization algorithm (AEDHOA), drawing inspiration from human hiking behavior. The algorithm introduces four strategies to improve population diversity and convergence efficiency, effectively tackling high-dimension feature selection problems while demonstrating strong global search capabilities and robustness [18].These studies suggest that biomimetic intelligent optimization algorithms are powerful tools for addressing curve and surface degree reduction in CAGD.

Specifically, Hu et al. applied the grey wolf optimization algorithm (GWO)—a bio-inspired swarm intelligence method—to investigate the multi-degree reduction of SG-Bézier curves [19]. Guo et al. employed a skewed normal cloud-modified whale optimization algorithm (WOA) to enhance the degree reduction performance for S-λ curves, leveraging its global search capabilities [20]. Additionally, Hu et al. proposed an enhanced golden jackal optimization algorithm (EGJO), which integrates oppositional learning, adaptive mutation, and binomial crossover strategies to improve the shape optimization of complex CSGC–Ball surfaces [21]. These studies collectively demonstrate the significant potential of biomimetic intelligent optimization algorithms in addressing complex curve and surface approximation problems, offering efficient and reliable solutions for high-order degree reduction tasks. However, existing biomimetic intelligent optimization algorithms still exhibit certain limitations when addressing degree reduction of complex curves. For instance, the grey wolf optimization algorithm (GWO) excels in global search capabilities but tends to get trapped in local optima in high-dimension problems and suffers from slow convergence [19]; the whale optimization algorithm (WOA) lacks sufficient local exploitation ability, particularly showing unstable performance in multimodal optimization problems [20]; and golden jackal optimization (GJO) struggles to maintain population diversity, often leading to premature convergence [21]. These shortcomings constrain the effectiveness of existing algorithms in complex curve degree reduction problems.

This paper addresses the limitations of the coati optimization algorithm [22], such as insufficient population diversity, limited local convergence capability, and a tendency to become trapped in local optima in complex optimization problems, by proposing a multi-strategy enhanced coati optimization algorithm (MSECOA) and applying it to the degree reduction approximation problem of Said–Ball curves. The main contributions of this paper are as follows:

- **Construction of a Said–Ball curve degree reduction model:** A new degree reduction approximation model for Said–Ball curves is proposed, with the objective function designed to minimize Euclidean distance and curvature error.
- **Proposal of a multi-strategy enhanced coati optimization algorithm:** The initial population distribution is optimized using a hybrid oppositional learning strategy based on a good point set, while global search and local exploitation capabilities are enhanced through a fitness–distance balance strategy and a dynamic spiral search strategy. An adaptive differential evolution mechanism is introduced to improve convergence speed and robustness. Comparative experiments on the IEEE CEC2017 and CEC-2020 test function suites, as well as four engineering constrained design problems, validate the MSECOA’s significant advantages in optimization accuracy, convergence speed, and robustness.
- **Validation of the MSECOA’s experimental performance in Said–Ball curve degree reduction:** Through numerical examples involving four different degree reduction levels, the MSECOA’s efficiency in multi-degree reduction approximation of Said–Ball curves is demonstrated. Experimental results show that the MSECOA effectively preserves the geometric features of curves while reducing their degree, offering an efficient solution for complex curve degree reduction problems.

The structure of this paper is organized as follows: Section 2 systematically elaborates degree reduction approximation problem of Said–Ball curves and constructs a degree reduction optimization model. Section 3 provides a detailed introduction to core principles and mathematical formulation of the coati optimization algorithm (COA). Section 4 presents a multi-strategy enhanced coati optimization algorithm (MSECOA), including pseudocode, flowcharts, and a theoretical analysis of its computational complexity. Section 5 comprehensively evaluates the MSECOA’s optimization performance using IEEE CEC2017 and CEC2022 benchmark functions. Section 6 applies the MSECOA to three typical engineering constrained optimization problems to verify its practical effectiveness. Section 7 validates the MSECOA’s superior performance in Said–Ball curve degree reduction through four sets of numerical experiments with varying degree reduction levels. Finally, Section 8 summarizes research contributions and innovations of this study and provides an outlook on future research directions.

## 2. Degree Reduction Approximation for Said–Ball Curves

### 2.1. Said–Ball Curves

Said–Ball curves, introduced by Said in 1991, represent an important class of parametric curves in computer-aided geometric design (CAGD). As a generalized form of Ball curves, they not only preserve many desirable properties of Bézier curves but also demonstrate superior flexibility and computational efficiency in specific applications. These curves are mathematically defined through control points and basis functions, enabling the precise representation of complex geometric shapes. Their applications span diverse engineering fields including mechanical design, aerospace engineering, and architectural modeling.

**Definition 1.**  *Given a set of control points $P_{i}$ (i=0,1,…,n) and the parameter t∈[0,1], the Said–Ball curve is expressed as Equation (1):*

(1) $$C\left(t\right)=\sum_{i=0}^{n}P_{i}S_{i}^{n}\left(t\right),$$

*where $S_{i}^{n}\left(t\right)$ denotes the Said–Ball basis function defined by Equation (2):*

(2) Sin(t)=⌊n/2⌋+iiti(1−t)⌊n/2⌋+1,0≤i≤⌊n/2⌋−1n⌊n/2⌋t⌊n/2⌋(1−t)⌊n/2⌋,i=⌊n/2⌋Sn−in(1−t),⌊n/2⌋+1≤i≤n

*here, ⌊·⌋ and ⌈·⌉ denote the floor and ceiling functions, respectively.*

### 2.2. Degree Reduction Problem of Said–Ball Curves

The degree reduction approximation problem of Said–Ball curves refers to reducing a high-degree Said–Ball curve $P_{n}\left(t\right)$ to a lower-degree Said–Ball curve $Q_{m}\left(t\right)$ (where m<n), while preserving the geometric features of the original curve as much as possible. The primary objective of degree reduction is to decrease the number of control points, thereby reducing computational complexity and storage requirements, while maintaining the shape accuracy of the curve.

**Definition 2.**  *The degree reduction problem of Said–Ball curves can be described as follows: Given an n-th degree Said–Ball curve, as shown in Equation (3),*

(3) $$P_{n}\left(t\right)=\sum_{i=0}^{n}P_{i}S_{i}^{n}\left(t\right),$$

*where $P_{i}$ are the control points and $S_{i}^{n}\left(t\right)$ are the Said–Ball basis functions, the objective is to determine a set of real numbers ${\left\{Q_{i}\right\}}_{i=0}^{m}$ such that the corresponding m-th degree Said–Ball curve*

(4) $$Q_{m}\left(t\right)=\sum_{i=0}^{m}Q_{i}S_{i}^{m}\left(t\right)$$

*satisfies the following condition over the closed interval [0,1]:*

(5) $$d\left(P_{n}\left(t\right),Q_{m}\left(t\right)\right)=\max_{0\leq t\leq 1}∥P_{n}\left(t\right)-Q_{m}\left(t\right)∥=\min.$$

*here, $d\left(P_{n}\left(t\right),Q_{m}\left(t\right)\right)$ represents the maximum error between the two curves, serving as the primary optimization objective of degree reduction problem.*

**Figure 1:**
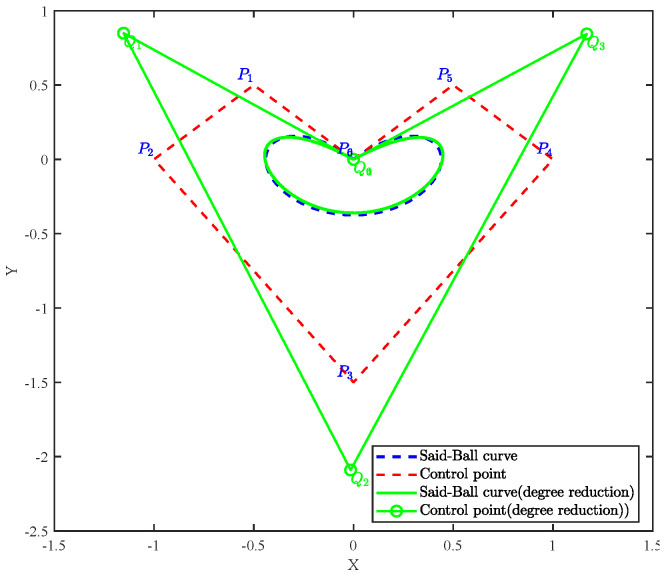
Comparison of 6th-degree Said–Ball curve before and after degree reduction.

Figure 1 illustrates a comparison between a sixth-degree Said–Ball curve and its reduction to a fourth-degree Said–Ball curve.

To preserve the geometric features of the original curve as much as possible during the degree reduction process, the following constraints are typically required to be satisfied:

1. End Point Constraint
  The reduced-degree curve $Q_{m}\left(t\right)$ must coincide with the original curve $P_{n}\left(t\right)$ at the end points, i.e.,
  (6) $$P_{n}\left(0\right)=Q_{m}\left(0\right)\;\mathrm{and}\;P_{n}\left(1\right)=Q_{m}\left(1\right).$$
  This constraint ensures consistency between the reduced-degree curve and the original curve at the start and end points.
2. Higher-Order Derivative Constraint
  To further maintain the smoothness of the curve, the higher-order derivatives of the reduced-degree curve $Q_{m}\left(t\right)$ at the end points should match those of the original curve, i.e.,
  (7) $$\begin{array}{c} \frac{d^{j}P_{n}\left(t\right)}{dt^{j}}{|}_{t=0}=\frac{d^{j}Q_{m}\left(t\right)}{dt^{j}}{|}_{t=0},\;j=0,1,\ldots ,r,\\ \frac{d^{j}P_{n}\left(t\right)}{dt^{j}}{|}_{t=1}=\frac{d^{j}Q_{m}\left(t\right)}{dt^{j}}{|}_{t=1},\;j=0,1,\ldots ,s, \end{array}$$
  where *r* and *s* denote the derivative orders at the start and end points, respectively, satisfying r+s+1≤m. A curve $Q_{m}\left(t\right)$ meeting these conditions is referred to as an end point-preserving (r,s)-order interpolation degree reduction approximation of $P_{n}\left(t\right)$.
3. Boundary Constraint
  The reduced-degree curve $Q_{m}\left(t\right)$ should lie within the convex hull of the original curve $P_{n}\left(t\right)$ to ensure reasonableness of the geometric shape. This constraint prevents potential shape distortion issues during the degree reduction process.

## 3. Coati Optimization Algorithm

The coati optimization algorithm (COA), a novel swarm intelligence optimization method, was proposed by Dehghani M et al. in 2023, drawing inspiration from the natural predatory behavior of coatis hunting iguanas and evading predators [22]. By simulating the hunting and escaping mechanisms of coatis, this algorithm exhibits strong global search capabilities and rapid convergence. However, the COA faces limitations in practical applications: uneven population distribution during the initialization phase may result in inadequate coverage of the search space, while its tendency to become trapped in local optima when addressing multimodal optimization problems can lead to a reduced convergence speed. These shortcomings constrain the algorithm’s performance in complex optimization scenarios.

### 3.1. Population Initialization

Coatis often operate in groups, with each coati’s position representing a candidate solution to the optimization problem. Since the positions of the coatis are random, the population must first be initialized, as shown in Equation (8):

(8) $$X_{i}:x_{i,j}=lb_{j}+r\;\cdot \;\left(ub_{j}-lb_{j}\right),i=1,2,\ldots ,N;j=1,2,\ldots ,m$$

where $X_{i}$ is the position of the *i*-th coati in the search space, $x_{i,j}$ is the value of the *j*-th decision variable, *N* is the number of coatis, *m* is the number of decision variables, *r* is a random real number in interval [0,1], and $lb_{j}$ and $ub_{j}$ are the lower and upper bounds of the *j*-th decision variable, respectively.

### 3.2. Hunting and Attack Strategy on Iguanas (Exploration Phase)

**Figure 2:**
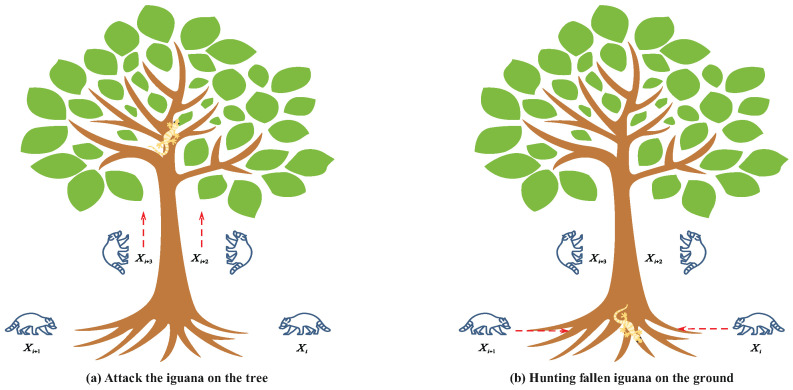
Hunting and attack strategy on iguanas (exploration phase).

In the exploration phase, the COA simulates the process of coatis hunting and attacking iguanas. Specifically, half of coatis climb trees to approach and intimidate iguana, while the other half remain on the ground waiting. When the iguana falls from the tree, ground-based coatis quickly kill it. This strategy enables coatis to migrate to different positions within the search space, emphasizing the COA’s global exploration capability in the problem-solving domain. Figure 2 illustrates this strategy in detail.

In COA design, the position of the individual with the best fitness in the coati population is assumed to represent the iguana’s position. Thus, the position update for tree-climbing coatis tasked with attacking iguanas is given by Equation (9):

(9) $$X_{i}^{P1}:x_{i,j}^{P1}=x_{i,j}+rand\left(0,1\right)\;\cdot \;\left(G_{j}-I\;\cdot \;x_{i,j}\right),i=1,2,\ldots ,\left[\frac{N}{2}\right];j=1,2,\ldots ,m$$

where $X_{i}^{P1}$ is the new position calculated for the *i*-th coati, $x_{i,j}^{P1}$ is its value in the *j*-th dimension, rand(0,1) is a random variable in [0,1], $G_{j}$ denotes the iguana’s position in the *j*-th dimension, and *I* is an integer randomly selected from the set {1,2}.

When the iguana falls to the ground, its position may land at a random location within the search space, with the position update formula given by Equation (10):

(10) $$G^{g}:G_{j}^{g}=ub_{j}+rand\left(0,1\right)\left(ub_{j}-lb_{j}\right)$$

Meanwhile, the movement of ground-based coatis in the search space follows a similar position update rule, as shown in Equation (11):

(11) $$\begin{array}{c} X_{i}^{P1}:x_{i,j}^{P1}=\left\{\begin{array}{c} x_{i,j}+rand\left(0,1\right)\;\cdot \;\left(G_{j}^{g}-Ix_{i,j}\right),\;F_{G,j}^{g}\leq F_{i,j}\\ x_{i,j}+rand\left(0,1\right)\;\cdot \;\left(x_{i,j}-G_{j}^{g}\right),\;\mathrm{otherwise} \end{array}\right.\\ i=[N/2]+1,[N/2]+2,\ldots ,N,\mathrm{and}j=1,2,...,m \end{array}$$

where $G_{j}^{g}$ represents the iguana’s position on the ground and *F* is the value of the objective function.

If a coati’s new position improves objective function value, that position is accepted; otherwise, the coati remains at its original position. This update condition is applied to all coatis i=1,2,…,N, as simulated by Equation (12):

(12) $$X_{i}=\left\{\begin{array}{cc} X_{i}^{P1}, & F_{i}^{P1}\leq F_{i}\\ X_{i}, & \mathrm{otherwise} \end{array}\right.$$

Through these strategies, the COA achieves efficient global exploration and local exploitation in the search space, resulting in superior performance in optimization problems.

### 3.3. Escape from Predators (Exploitation Phase)

**Figure 3:**
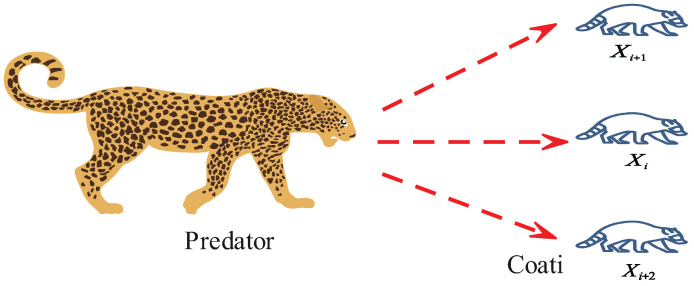
Process of escaping predators (exploitation phase).

As shown in Figure 3, the second phase of the COA simulates the natural behavior of coatis escaping from predators through mathematical modeling, demonstrating the algorithm’s local search capability. In this phase, coatis move toward safer nearby positions to evade potential threats.

To precisely model this behavior, a random position within each coati’s neighborhood is generated according to Equation (13), guiding coati to migrate toward that position. This strategy not only enhances the algorithm’s fine-grained search capability within local regions but also significantly improves the solution accuracy and convergence efficiency.

(13) $$\begin{array}{c} X_{i}^{P2}:x_{i,j}^{P2}=x_{i,j}+\left(1-2r\right)\;\cdot \;\left(lb_{j}^{local}+r\;\cdot \;\left(ub_{j}^{local}-lb_{j}^{local}\right)\right)\\ lb_{j}^{local}=\frac{lb_{j}}{t},ub_{j}^{local}=\frac{ub_{j}}{t},\mathrm{where}\;t=1,2,\ldots ,T.\\ i=1,2,\ldots ,N;j=1,2,\ldots ,m. \end{array}$$

Here, $lb_{j}^{local}$ and $ub_{j}^{local}$ are the local lower and upper bounds of the *j*-th decision variable, respectively; $lb_{j}$ and $ub_{j}$ are the global lower and upper bounds of the *j*-th decision variable; *t* is the iteration counter; $X_{i}^{P2}$ is the new position of the *i*-th coati in the second phase; and $x_{i,j}^{P2}$ is its value in the *j*-th dimension. If the newly computed position improves the objective function value, it is accepted; otherwise, the coati remains at its original position. This condition is expressed by Equation (14):

(14) $$X_{i}=\left\{\begin{array}{cc} X_{i}^{P_{2}}, & F_{i}^{P2}⩽F_{i}\\ X_{i}, & \mathrm{otherwise} \end{array}\right.$$

where $F_{i}^{P2}$ is the fitness value of the new position and $F_{i}$ is the fitness value of the current position.

Through simulating the hunting and escape behaviors of coatis, the COA updates the coati positions in two phases, iterating continuously until the maximum number of iterations is reached. Upon completion, the algorithm outputs the best solution as the result. By integrating global exploration and local exploitation, the COA exhibits efficient optimization search capabilities, providing robust support for solving complex problems.

## 4. Multi-Strategy Enhanced Coati Optimization Algorithm (MSECOA)

### 4.1. Good Point Set Hybrid Oppositional Learning Strategy

In optimization algorithms, the quality of the initial population significantly impacts algorithm performance. Traditional random initialization methods often produce unevenly distributed solutions, leading to local clustering and reduced global search capability. To address this, a population initialization method based on good point set sequences and a hybrid oppositional learning strategy is proposed in this paper. This approach combines the uniform distribution properties of good point sets with the diversity-enhancing mechanism of hybrid oppositional learning to generate an initial population that is both uniformly distributed and highly diverse, effectively resolving the issues of uneven distribution and local clustering inherent in traditional methods.

#### 4.1.1. Good Point Set Sequence

To overcome limitations of traditional random initialization, a good point set (GPS) is introduced as a foundational tool for population initialization in this study. The good point set is a low-discrepancy sequence generation method rooted in number theory, capable of producing uniformly distributed point sets in high-dimension spaces [23]. Unlike pseudo-random number generation methods, GPS leverages properties of prime numbers and trigonometric functions to ensure that the generated points exhibit low discrepancy, thereby avoiding local clustering in good point set initial population.

**Definition 3.**  *Let $G_{s}$ denote the unit cube in good point set s-dimension Euclidean space, and let $r\in G_{s}$. If the point set $p_{n}\left(k\right)=\left\{\left(\left\{k\;\cdot \;r_{1}^{\left(n\right)}\right\},\ldots ,\left\{k\;\cdot \;r_{s}^{\left(n\right)}\right\}\right),1\leq k\leq n\right\}$ has a discrepancy satisfying $\phi \left(n\right)=C\left(r,\varepsilon \right)\;\cdot \;n^{-1+\varepsilon }$ (where C(r,ε) is a constant dependent only on r and ε, and ε is any positive number), then $p_{n}\left(k\right)$ is called a good point set and r is termed a good point.*

*The specific construction method is as follows: set $\left\{r_{k}=2\cos\left(2\pi k/p\right),1\leq k\leq s\right\}$, where p is the smallest prime number satisfying (p−3)/2≥s and $\left\{k\;\cdot \;r_{i}^{\left(n\right)}\right\}$ represents the fractional part of $k\;\cdot \;r_{i}^{\left(n\right)}$.*

Specifically, initial solutions are generated using the good point set as shown in Equation (15):

(15) $$x_{i,j}=lb_{j}+\left(\cos\left(2\pi \;\cdot \;\frac{i}{p_{j}}\right)\;\mathrm{mod}\;1\right)\;\cdot \;\left(ub_{j}-lb_{j}\right)$$

where $p_{j}$ is prime number corresponding to the *j*-th dimension and $\left[lb_{j},ub_{j}\right]$ denotes good point set lower and upper bounds of the search space. This method ensures uniform coverage of the initial population across the search space while enhancing the algorithm’s global search capability.

**Figure 4:**
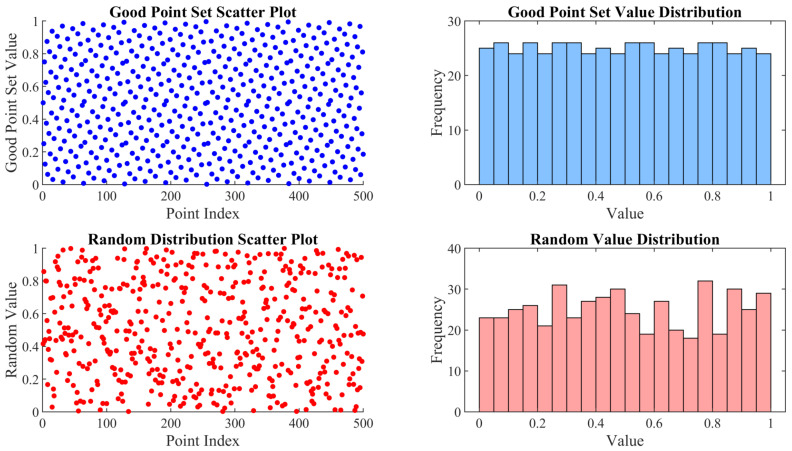
Comparison of population distribution between the good point set and random methods.

Figure 4 compares the individual distributions generated by the good point set and random methods. The good point set yields a uniform distribution with broad coverage and no clustering, enabling the comprehensive traversal of the solution space, whereas the random method results in uneven distribution prone to local optima. The stable construction of the good point set, independent of dimensionity, produces high-quality initial solution sets, significantly improving the algorithm’s global search capability and convergence efficiency.

#### 4.1.2. Hybrid Oppositional Learning Strategy

**Figure 5:**
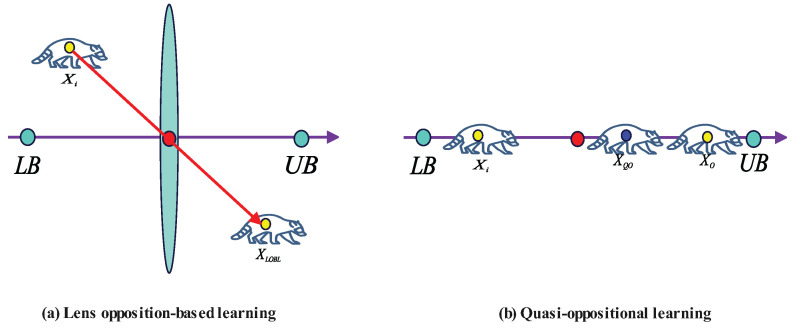
Schematic of hybrid oppositional learning.

In complex optimization problems, although the good point set generates a uniformly distributed initial population, relying solely on uniform distribution may not suffice to fully explore the entire search space. To address this, a hybrid oppositional learning strategy [24] is introduced in this study, dynamically selecting oppositional learning methods via a random switching mechanism to effectively enhance population diversity and search capability, as illustrated in Figure 5.

The hybrid oppositional learning strategy encompasses two mechanisms: lens oppositional learning and quasi-oppositional learning. Lens oppositional learning generates an opposite solution to the current solution through symmetric mapping, as expressed by

(16) $$X_{\mathrm{opp}}=lb+ub-X$$

where *X* is the current solution, $X_{\mathrm{opp}}$ is its opposite solution, and lb and ub are the lower and upper bounds of the search space, respectively. The advantage of lens oppositional learning lies in its ability to rapidly generate solutions symmetric to the current one, thereby expanding the search range and enhancing global exploration.

Quasi-oppositional learning introduces random perturbations between the current solution and its opposite to generate new solutions, as given by

(17) $$X_{\mathrm{qopp}}=X+\alpha \;\cdot \;\left(X_{\mathrm{opp}}-X\right)$$

where α is a random perturbation factor, typically a random number in [0,1]. The strength of quasi-oppositional learning is its capacity to produce solutions between the current and opposite solutions, enhancing local search capability and improving convergence efficiency.

To leverage the strengths of both methods, a hybrid strategy is further proposed. This strategy employs a random switching probability *p* to dynamically select between lens oppositional learning and quasi-oppositional learning for generating new solutions. Specifically, a random number r∈[0,1] is generated, and the choice of oppositional learning method is determined based on the relationship between *r* and *p*:

(18) $$X_{\mathrm{new}}=\left\{\begin{array}{cc} lb+ub-X, & \mathrm{if}\;r<p\\ X+\alpha \;\cdot \;(lb+ub-2X), & \mathrm{else} \end{array}\right.$$

where α∼U(0,1) is a perturbation factor randomly drawn from a uniform distribution. When r<p, lens oppositional learning is used to generate the opposite solution; when r≥p, quasi-oppositional learning is applied to produce a quasi-opposite solution. This probability-based switching strategy balances global exploration and local exploitation, effectively preventing the algorithm from being trapped in local optima. By dynamically adjusting the search direction, lens oppositional learning enhances global exploration, while quasi-oppositional learning boosts local exploitation efficiency, enabling the algorithm to better balance exploration and exploitation in complex multimodal optimization problems and avoid premature convergence.

### 4.2. Fitness–Distance Balance Strategy

In the COA, positions are updated through random subjects during hunting and attacking phases, with optimization performance relying on balancing exploration and exploitation. During optimization, the algorithm must maintain global search capability to identify promising regions in solution space while performing refined searches in the neighborhoods of high-quality solutions to enhance convergence accuracy. The fitness–distance balance (FDB) strategy is a novel selection approach designed to choose candidate solutions that contribute most to search process [25]. Unlike traditional selection methods, FDB considers not only the fitness value of a candidate solution but also its distance from the optimal solution to compute a score. This dual criterion ensures that the candidate with the highest score is selected, efficiently guiding the population search while avoiding individuals too close to the optimal solution, thus preventing entrapment in local optima. The mathematical model of the FDB strategy is as follows:

Let the current population be $P=\{X_{1},X_{2},\ldots ,X_{N}\}$, with the best individual denoted as $X_{best}$. The FDB score $S_{i}$ for other individuals $X_{i}$ is calculated as shown in Equation (19):

(19) $$S_{i}=\omega^{*}\;\cdot \;\left(\frac{f\left(X_{i}\right)-f_{\min}}{f_{\max}-f_{\min}}\right)+\left(1-\omega^{*}\right)\;\cdot \;\left(\frac{D_{\max}-D\left(X_{i},X_{best}\right)}{D_{\max}-D_{\min}}\right)$$

where $f(X_{i})$ is the fitness value of the individual $X_{i}$, $f_{\min}$ and $f_{\max}$ are the minimum and maximum fitness values in the current population, $D(X_{i},X_{best})$ is the Euclidean distance between $X_{i}$ and $X_{best}$, $D_{\min}$ and $D_{\max}$ are the minimum and maximum distances in the current population, and $\omega^{*}$ is a weight within [0,1] that balances fitness and distance.

By weighting these two distances, the candidate solution with the highest score is selected to guide the algorithm’s search. In this study, the FDB strategy is applied to the exploration phase of the COA, modifying the selection strategy for $G_{j}$ individuals across both phases to use FDB, thereby selecting the most representative individuals for position updates.

### 4.3. Dynamic Spiral Search

In the exploitation phase of the COA, the search space gradually narrows as iterations progress, weakening the ability to explore unknown regions. To address this, a dynamic spiral search method inspired by the spiral search mechanism of the whale optimization algorithm (WOA) [26] is introduced. This method dynamically adjusts the spiral shape parameter based on the iteration count, enhancing global search capability and optimization accuracy. Originally derived from humpback whale predation behavior, the spiral search mechanism narrows encirclement via a spiral path to approach prey. In traditional spiral update models, the spiral shape parameter is constant, accelerating late-stage convergence but often resulting in a singular search path and local optima entrapment. By introducing an iteration parameter to dynamically adjust the spiral shape, this issue is effectively mitigated, with the update formula given by Equation (20):

(20) $$\begin{array}{c} z=e^{k\;\cdot \;\cos(\frac{\pi \;\cdot \;t}{T})}\\ X\left(t+1\right)=e^{zl}\;\cdot \;\cos\left(2\pi l\right)\;\cdot \;r\;\cdot \;\left(X_{best}\left(t\right)-rand\;\cdot \;X_{best}\left(t\right)\right) \end{array}$$

where *k* is a constant defining the logarithmic spiral shape, *l* is a random number between [−1,1], *t* is the current iteration, *T* is the maximum iteration count, and *r* is the sensitivity range of each coati.

The position update for coatis in the exploitation phase is thus defined by Equation (21):

(21) $$X_{i}^{P2}=\left\{\begin{array}{cc} x_{i,j}^{P2}=e^{zl}\;\cdot \;\cos\left(2\pi l\right)\;\cdot \;r\;\cdot \;\left(X_{best}\left(t\right)-rand\;\cdot \;X_{best}\left(t\right)\right), & \mathrm{if}\;F_{i}^{P1}⩽mean\left(F\right)\\ x_{i,j}^{P2}=x_{i,j}+\left(1-2r\right)\;\cdot \;\left(lb_{j}^{local}+r\;\cdot \;\left(ub_{j}^{local}-lb_{j}^{local}\right)\right), & \mathrm{else} \end{array}\right.$$

Thus, in early iterations, the COA employs a larger spiral shape for the global search to accelerate convergence; in later iterations, the spiral shape is gradually reduced as the iteration count increases, enabling refined searches to enhance optimization accuracy.

### 4.4. Integration of Self-Adaptive Differential Evolution Algorithm

The differential evolution algorithm (DE) optimizes population individuals through mutation, crossover, and selection operations, using differential vectors to generate new individuals and improve search efficiency. In the MSECOA, the differential mutation mechanism of DE is incorporated into the COA, leveraging differential vector generation to enhance solution space exploration and avoid local optima. Additionally, the self-adaptive differential evolution algorithm (SADE) [27] is integrated, introducing an adaptive scaling factor and linearly decreasing crossover probability to dynamically adjust exploration and exploitation capabilities. This enhances the global search in early stages and local precision in later stages, further improving algorithm performance. The mathematical models for each step are described below:

The mutation operation generates a step size by randomly selecting two individuals from the population and using their difference vector, as shown in Equation (22):

(22) $$y_{i,j}=x_{i,j}+F\;\cdot \;\left(x_{R1,j}-x_{R2,j}\right),\;i=1,2,3,\ldots ,N$$

where $X_{i}$ is *i*-th individual in population, $x_{R1}$ and $x_{R2}$ are two distinct individuals randomly chosen from the population, *j* is the *j*-th dimension of the solution vector, and *F* is the scaling factor controlling the mutation step size.

The crossover operation generates a trial individual by combining information from target and mutated individuals, as expressed in Equation (23):

(23) $$Z_{i}:z_{i,j}=\left\{\begin{array}{cc} y_{i,j}, & r<CR\;||\;j_{r}=j\\ x_{i,j}, & \mathrm{otherwise} \end{array}\right.$$

where CR is the crossover probability, *r* is a random number, and $j_{r}$ is a randomly selected dimension index.

The selection operation determines whether the target individual is updated by comparing the fitness values of trial and target individuals, as given by Equation (24):

(24) $$X_{i}=\left\{\begin{array}{cc} Z_{i}, & F\left(Z_{i}\right)<F\left(X_{i}\right)\\ X_{i}, & \mathrm{otherwise} \end{array}\right.$$

where $F(Z_{i})$ and $F(X_{i})$ are the fitness values of trial and target individuals, respectively.

SADE further enhances performance by adaptively adjusting the scaling factor *F* and crossover probability CR, as implemented in Equation (25):

(25) $$\begin{array}{c} F=0.5\times (1+rand())\\ CR=0.9-\left(0.9-0.1\right)\times \frac{t}{T} \end{array}$$

where rand() generates a random number in [0,1], *t* is the current iteration, and *T* is the maximum iteration count.

### 4.5. Flowchart and Pseudocode of MSECOA

**Figure 6:**
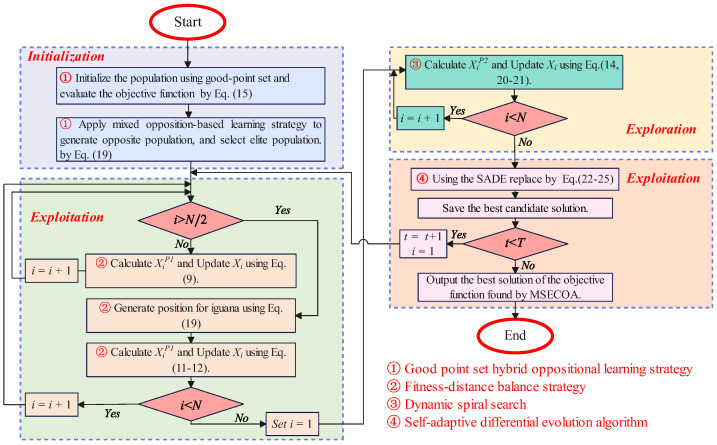
Flowchart of MSECOA.

The pseudocode of the MSECOA is provided in Algorithm 1, while the process of the algorithm is described in Figure 6.
**Algorithm 1** MSECOA: Multi-Strategy Enhanced Coati Optimization Algorithm**Require:** Population size *N*, dimensionity of problem dim, Maximum number of iterations *T*.**Ensure:** Best objective function value fbest, Best solution position Xbest, Convergence curve $COA_{curve}$.  1:**Initialize population:** using Good Point Set and Opposition Learning  2:**for** i=1 to *N* **do**  3:    X(i,:)←initializePopulation(), fit(i)←fitness(X(i,:))  4:**end for**  5:**for** t=1 to *T* **do**  6:    Update global best solution: Xbest,fbest  7:    **Exploration Phase:** Use fitness–distance balance to guide hunting and attacking strategy  8:    **for** i=1 to N/2 **do**  9:         Calculate new position for *i*-th coati using Equation (19).10:         Update position of *i*-th coati using Equation (12).11:    **end for**12:    **for** i=N/2 to *N* **do**13:        Calculate new position for *i*-th coati using Equation (19).14:        Update position of *i*-th coati using Equation (12).15:    **end for**16:    **Exploitation Phase:** Use Dynamic Spiral Search17:    **for** i=1 to *N* **do**18:        Calculate new position for *i*-th coati using Equation (21).19:        Update position of *i*-th coati using Equation (14).20:    **end for**21:    **Adaptive Differential Evolution:** Apply mutation, crossover, and selection22:    **for** i=1 to *N* **do**23:        Calculate new position for *i*-th coati using Equations (22)–(25).24:        Update position of *i*-th coati using Equation (14).25:    **end for**26:    Record best-so-far fitness: $COA_{curve}\left(t\right)\leftarrow fbest$27:**end for**28:**return** fbest, Xbest

### 4.6. Time Complexity Analysis

Time complexity serves as a critical metric for evaluating algorithm performance, primarily determined by three core processes: population initialization, fitness evaluation, and position updating. The original COA exhibits a time complexity of O(N×D×T), where *N*, *D*, and *T* represent the population size, problem dimensionity, and maximum iterations, respectively. The MSECOA’s complexity analysis reveals the following:

1. Good Point Set with Opposition-Based Learning: Initialization requires O(N×D) operations, while opposition-based component adds another O(N×D) computation. This combined strategy maintains an initialization complexity of O(N×D) without substantial overhead.
2. Fitness–Distance Balance Strategy: Each iteration computes distances between individuals and the global best (O(N×D)) and performs selection operations. This single traversal operation preserves overall O(N×D×T) complexity.
3. Dynamic Spiral Search: Exploitation-phase spiral search operates within O(N×D) complexity. As it merely modifies the position update equations without additional loops, it imposes a negligible computational burden.
4. Adaptive Differential Evolution: Mutation–crossover–selection operations in each iteration require O(N×D) computations. Executing in parallel with the COA’s native updates, they maintain original O(N×D×T) complexity.

Notably, the MSECOA retains base O(N×D×T) complexity while achieving superior optimization performance through these strategic enhancements.

## 5. Experimental Validation and Result Analysis

### 5.1. Experimental Design and Parameter Settings

The simulation test environment for this section’s experiments is based on the Windows 10 operating system, with hardware consisting of an AMD Ryzen 7 4800H processor running at a base frequency of 2.90 GHz. All algorithm implementations and tests were conducted on the MATLAB 2022b platform.

To validate the effectiveness of the MSECOA, performance tests were conducted using a total of 41 test functions from IEEE CEC2017 [28] and CEC2022 [29]. In experiments, the MSECOA was compared against ten algorithms: the basic COA, the improved COA algorithm (ICOA [30]), popular optimization algorithms and their variants (whale optimization algorithm (WOA) [26], the Arithmetic Optimization Algorithm (AOA) [31], Nonlinear Chaotic Harris Hawks Optimization (NCHHO) [32], Improved Sand Cat Swarm Optimization (ISCSO) [33]), newly proposed algorithms from 2024 (Black-winged Kite Algorithm (BKA) [34], the hiking optimization algorithm (HOA) [35]), and a top-performing CEC algorithm (LSHADE_cnEpSin [36]). For the experimental setup, the maximum number of iterations for each algorithm was set to 1000, the population size was uniformly fixed at 30, and each algorithm was independently run 30 times.

Furthermore, mean (Mean) and standard deviation (Std) were selected as the evaluation metrics, and the experimental results were subjected to the Friedman test [37] and Wilcoxon rank-sum test to verify the MSECOA’s effectiveness. The Wilcoxon rank-sum test results were derived from *p*-values obtained under a significance level of α=0.05. In this testing framework, “+” indicates that the MSECOA outperforms the compared algorithm in terms of performance, “=” signifies no significant difference between the MSECOA and compared algorithm, and “−” denotes that the compared algorithm surpasses the MSECOA in performance. The parameter settings for these algorithms are detailed in Table 1.

### 5.2. Ablation Study of MSECOA

To rigorously evaluate the contribution of each enhancement strategy in the MSECOA, we conducted a systematic ablation study. The experiments were carried out on the IEEE CEC2017 benchmark suite, which comprises 29 test functions categorized into four types: unimodal (F1–F3), multimodal (F4–F10), hybrid (F11–F20), and composite functions (F21–F30). A detailed description of these functions, including their theoretical optimal values, is provided in Table 2.

To assess the individual effectiveness of each enhancement strategy, we compared the MSECOA with four variant algorithms, each incorporating only one of the proposed strategies:

- **GCOA**: COA with a good point set hybrid oppositional learning strategy.
- **FCOA**: COA with a fitness–distance balance strategy.
- **DCOA**: COA with a dynamic spiral search strategy.
- **SCOA**: COA with a self-adaptive differential evolution mechanism.

**Figure 7:**
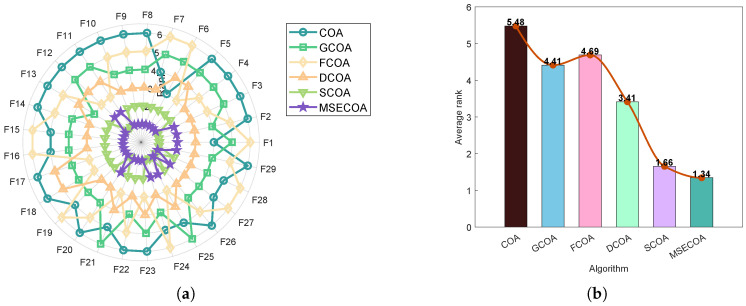
The ablation study on CEC2017 (10-dimension). (**a**) The radar chart. (**b**) The average rank chart.

The performance comparison is illustrated in Figure 7, which includes a radar chart and an average rank chart. The results clearly demonstrate that the MSECOA achieves the best overall performance, with the lowest average rank of 1.34, significantly outperforming all individual variants. In contrast, the FCOA exhibits the highest average rank of 4.69, indicating limited optimization capability when applied in isolation. The GCOA, DCOA, and SCOA achieve average ranks of 4.41, 3.41, and 1.66, respectively, reflecting their moderate but distinct contributions to algorithmic improvement.

These findings confirm that the integration of all four enhancement strategies—the good point set hybrid oppositional learning approach, fitness–distance balance mechanism, dynamic spiral search technique, and self-adaptive differential evolution algorithm—results in a synergistic effect. This combination significantly enhances the algorithm’s convergence speed, solution accuracy, and overall robustness.

### 5.3. Comparative Analysis of Algorithms on CEC2017 Benchmark

#### 5.3.1. Experimental Results and Analysis of CEC2017 Benchmark in Dimension 10

The performance of the different algorithms was evaluated using metrics such as mean and standard deviation, with detailed results presented in Table 3. To further validate the statistical significance of the performance differences between the algorithms, the Wilcoxon rank-sum test was conducted, and the results are provided in Table 4.

From Table 3, it is evident that the MSECOA performs exceptionally well on 17 benchmark test functions (F1, F3–F5, F7, F8, F11–F16, F18, F19, F23, F26, and F29), with both the mean and standard deviation of its optimal values surpassing those of the other eight compared algorithms. For functions F6, F9, F17, F20, F22, and F25, the mean optimal value of the MSECOA is second only to the best-performing algorithm; for F10, F24, F27, and F30, the MSECOA achieves the best mean value, though its standard deviation is not the smallest. According to the Friedman test results, the MSECOA ranks first, significantly outperforming other algorithms, with LSHADE_cnEpSin and the BKA ranking second and third, respectively, while the COA ranks ninth. The experimental results demonstrate that the MSECOA exhibits strong optimization capability on the CEC2017 test suite (dimension 10), particularly excelling in unimodal, multimodal, hybrid, and composition functions. Among the 29 test functions, the MSECOA achieves the best or near-best results on most, with particularly notable performance on high-dimension complex functions (e.g., F12, F18, F30). Although it slightly underperforms LSHADE_cnEpSin on some composition functions (e.g., F27, F28, F29), its overall performance remains significantly superior, with a Friedman rank of 1.207, securing the top position.

Based on the Wilcoxon rank-sum test results in Table 4, the MSECOA demonstrates a significant performance advantage on the CEC2017 test suite (dimension 10). Across the 29 benchmark test functions, the MSECOA significantly outperforms the ICOA, the COA, the WOA, the AOA, the HOA, NCHHO, and ISCSO on all 29 functions (*p* < 0.05), particularly excelling in unimodal functions (e.g., F1, F3–F5), multimodal functions (e.g., F7, F8, F11–F16), and high-dimension complex functions (e.g., F12, F18, F30). Compared to LSHADE_cnEpSin, the MSECOA is significantly superior on 23 functions, performs comparably on 4 functions (p≥0.05), and is slightly inferior on 2 functions (e.g., F9 and F23). Additionally, the MSECOA outperforms the BKA significantly on 22 functions, further confirming its robustness and stability. In summary, the MSECOA exhibits strong adaptability and generalization across diverse optimization problems, proving to be an efficient and stable algorithm, particularly suited for high-dimension complex optimization challenges.

**Figure 8:**
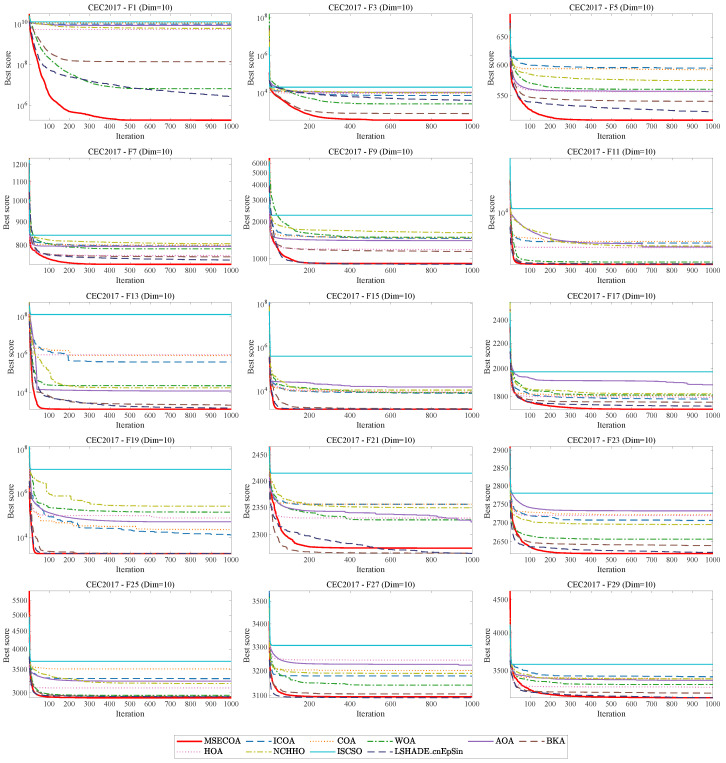
Convergence curves of different algorithms (CEC2017, dimension 10).

As shown in Figure 8, this paper presents the iterative convergence curves of different algorithms on 15 benchmark functions from the CEC2017 test suite. The experimental results indicate that the MSECOA significantly outperforms the other nine compared algorithms in terms of global search capability, convergence speed, and convergence accuracy when solving these functions. Specifically, the MSECOA demonstrates strong global exploration ability in early iterations, rapidly identifying potential optimal solution regions, while in later iterations, its local exploitation capability is fully exhibited, enabling convergence to the global optimum with high precision.

#### 5.3.2. Experimental Results and Analysis of CEC2017 Benchmark in Dimension 30

When the dimension is set to 30, the performance of each algorithm was evaluated based on the mean and standard deviation of its optimization results, with specific data presented in Table 5. Additionally, to further verify the statistical significance of the performance differences between the algorithms, the Wilcoxon rank-sum test results are provided in Table 6.

From the experimental results in Table 5, it is evident that the MSECOA exhibits a significant performance advantage on the CEC2017 test suite (dimension 30). Specifically, the MSECOA achieves the best mean values on 22 out of the 29 benchmark test functions, with particularly outstanding performance on high-dimension complex functions. Furthermore, the standard deviation of the MSECOA is significantly lower than that of the other algorithms on most functions, indicating high stability and robustness in its optimization outcomes. According to Friedman test results, the MSECOA ranks at 1.103, markedly outperforming the other compared algorithms, further confirming its superiority in high-dimension optimization problems. LSHADE_cnEpSin and the BKA rank second and third, respectively, showing relatively strong performance, though they fall short of the MSECOA on complex functions. In contrast, algorithms such as the ICOA, COA, WOA, and AOA perform poorly, especially on high-dimension complex functions.

Based on the Wilcoxon rank-sum test results in Table 6, the MSECOA demonstrates a significant performance advantage on the CEC2017 test suite (dimension 30). Across the 29 test functions, the MSECOA significantly outperforms the ICOA, the COA, the WOA, the AOA, the HOA, NCHHO, and ISCSO (*p* < 0.05), with particularly notable optimization capabilities on unimodal, multimodal, and high-dimension complex functions. Compared to LSHADE_cnEpSin, the MSECOA exhibits a significant advantage on 26 functions, performing comparably or slightly worse on a few composition functions. Additionally, the MSECOA significantly surpasses the BKA on 25 functions, further evidencing its exceptional robustness and stability. These results collectively demonstrate that the MSECOA possesses strong competitiveness across various types of optimization problems.

**Figure 9:**
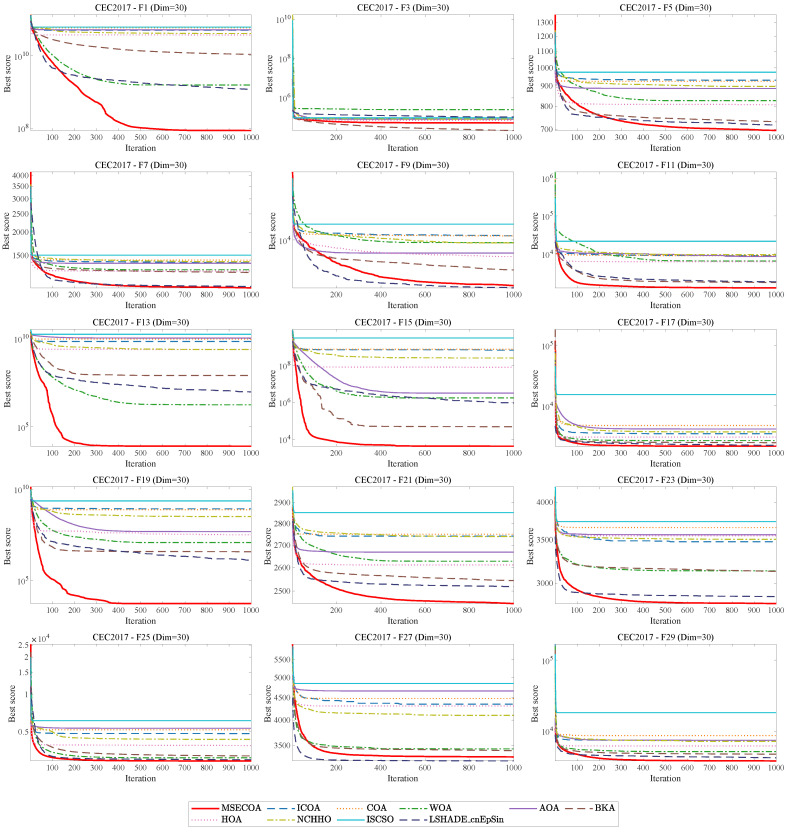
Convergence curves of different algorithms (CEC2017, dimension 30).

As shown in Figure 9, the MSECOA exhibits excellent performance in convergence speed, accuracy, and global search capability. In functions such as F1, F3, and F19, the MSECOA rapidly approaches the optimal solution, with the fitness values significantly lower than those of other algorithms. In multimodal functions (e.g., F7, F9), the MSECOA effectively avoids local optima, demonstrating strong global search capability. In contrast, algorithms such as the ICOA and COA show a clear gap in convergence speed and accuracy compared to the MSECOA.

### 5.4. Comparative Analysis of Algorithms on CEC2022 Benchmark

To further validate the performance of the MSECOA, the CEC2022 test suite was employed for experimental evaluation in this study. The IEEE CEC2022 test suite comprises four types of functions—unimodal (F1), basic (F2–F5), hybrid (F6–F8), and composition (F9–F12)—capable of comprehensively assessing algorithm performance across diverse optimization problems. The names and theoretical optimal values of each function are detailed in Table 7. Through experiments with this test suite, the adaptability and robustness of the MSECOA in handling varied optimization challenges can be more thoroughly evaluated.

#### Experimental Results and Analysis of CEC2022 Benchmark in Dimension 20

When the dimension is set to 20, metrics such as the mean and standard deviation of the experimental results are presented in Table 8, used to evaluate the performance of each algorithm on the CEC2022 test suite. Additionally, Table 9 provides the Wilcoxon rank-sum test results to further verify the statistical significance of the performance differences between algorithms, enabling a comprehensive analysis of their performance in high-dimension optimization problems.

From the experimental results in Table 8, it is evident that the MSECOA demonstrates a significant performance advantage on the CEC2022 test suite (dimension 20). Specifically, the MSECOA achieves the best mean values on multiple functions out of 12 test functions. Furthermore, the standard deviation of the MSECOA is significantly lower than that of the other algorithms on most functions, indicating high stability and robustness in its optimization outcomes. According to Friedman test results, the MSECOA ranks at 1.500, significantly outperforming the other compared algorithms, further confirming its superiority across diverse optimization problems. LSHADE_cnEpSin and the BKA rank second and third, respectively, exhibiting relatively strong performance, though they fall short of the MSECOA on complex functions. Other algorithms perform adequately on some functions but generally lag behind the MSECOA.

**Figure 10:**
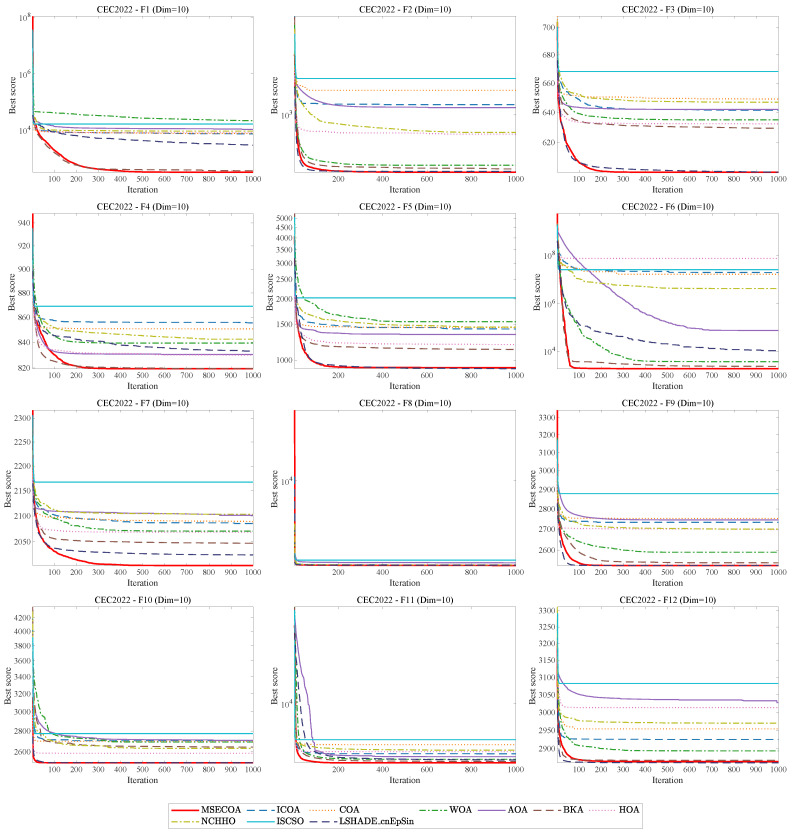
Convergence curves of different algorithms (CEC2022, dimension 20).

The Wilcoxon test results in Table 9 show that the MSECOA performs exceptionally on the CEC2022 suite (20-dimension). Across all 12 test functions, the MSECOA significantly outperforms the ICOA, the COA, the WOA, the AOA, the HOA, NCHHO, and ISCSO (*p* < 0.05). Compared to the BKA, the MSECOA is significantly superior on 10 functions, with equal performances on F2 and F4. Against LSHADE_cnEpSin, the MSECOA excels on seven functions, matches on two (F2 and F9), and is slightly inferior on one (F7). Overall, the MSECOA exhibits stronger optimization capability in 20-dimension problems. Additionally, Figure 10 illustrates convergence curves of different algorithms on CEC2022 benchmark functions.

The experimental results of various algorithms across different test sets are presented in Table 10.

As indicated by Table 10, the MSECOA exhibits superior stability and optimization precision across multi-dimension optimization problems. Its Friedman value and final ranking are notably better than those of other algorithms, reflecting an effective balance between search accuracy, computational efficiency, and stability. In contrast, algorithms such as the ICOA, COA, and WOA demonstrate poorer performance in high-dimension problems, revealing evident performance bottlenecks. Although the BKA and LSHADE_cnEpSin maintain consistent performance, their optimization effectiveness is limited in highly challenging test sets. With its robust performance, the MSECOA achieves a cumulative ranking of one, underscoring its efficiency and reliability in tackling complex optimization problems.

## 6. MSECOA for Engineering Constrained Design Problems

To evaluate the optimization performance of the MSECOA in addressing real-world engineering challenges, four classical engineering design problems—namely, three-bar truss design [38], welded beam design [39], compression spring design [40], and pressure vessel design [41]—are selected for testing. These problems vary in solution complexity and constraint conditions, providing a comprehensive assessment of the algorithm’s adaptability and robustness in intricate engineering scenarios. The experimental setup is configured with a population size of 30 and a maximum of 1000 iterations, while a penalty coefficient of $10^{10}$ is applied to handle constraints. To ensure the reliability of results, each algorithm is independently executed 30 times, with the best, worst, mean, and standard deviation of the objective function values recorded.

### 6.1. Three-Bar Truss Design

**Figure 11:**
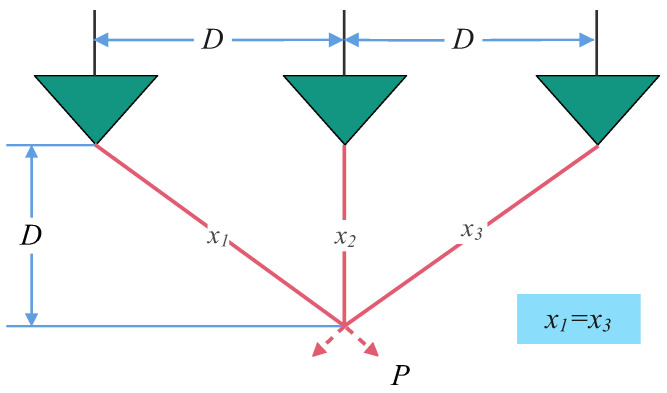
Schematic of the three-bar truss design problem.

The objective of three-bar truss design (TBT) problem [38] is to minimize the volume of truss by optimizing cross-sectional areas. A schematic of the three-bar truss design problem is illustrated in Figure 11.

The mathematical model for the TBT problem is presented in Equation (26).

(26) $$\begin{array}{c} Minf\left(x\right)=a\left(2\sqrt{2}x_{1}+x_{2}\right)\\ s.t\left\{\begin{array}{c} g_{1}=\frac{\sqrt{2}x_{1}+x_{2}}{\sqrt{2}{x_{1}}^{2}+2x_{1}x_{2}}P-\sigma \leq 0\\ g_{2}=\frac{x_{2}}{\sqrt{2}{x_{1}}^{2}+2x_{1}x_{2}}P-\sigma \leq 0\\ g_{3}=\frac{1}{\sqrt{2}x_{2}+x_{1}}P-\sigma \leq 0\\ a=100\mathrm{cm},P=2\mathrm{kN}/{\mathrm{cm}}^{2}\\ \sigma =2\mathrm{kN}/{\mathrm{cm}}^{2}\\ 0\leq x_{1},x_{2}\leq 1 \end{array}\right. \end{array}$$

The statistical results, including the optimal parameters and objective function values achieved by each algorithm for the three-bar truss design problem, are summarized in Table 11.

As evidenced in Table 11, the MSECOA demonstrates exceptional performance in the three-bar truss design problem. The best, mean, and standard deviation of the objective function values are recorded as 263.8958, 263.8958, and $2.98556\times 10^{-14}$, respectively, outperforming all competing algorithms and securing the top rank. Specifically, the optimal parameter combination obtained by the MSECOA is $\left\{x_{1}=0.7887,x_{2}=0.4082\right\}$, yielding a minimum objective function value that aligns with the theoretical optimum.

### 6.2. Welded Beam Design

**Figure 12:**
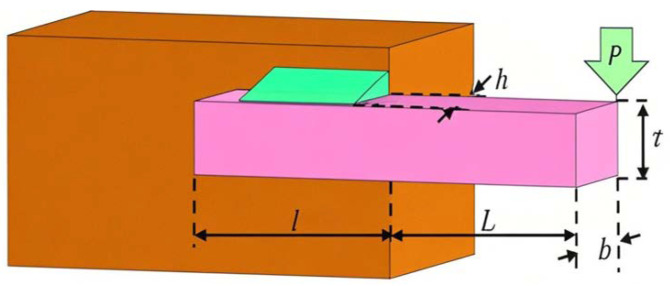
Schematic of welded beam design problem.

The objective of the welded beam design (WBD) problem [39] is to minimize the manufacturing cost of a welded beam by optimizing the beam length (*l*), height (*t*), thickness (*b*), and weld thickness (*h*) while satisfying constraints such as shear stress (τ), bending stress (θ), the buckling load of the beam ($P_{c}$), end deflection (δ), and boundary conditions. A schematic of the WBD problem is presented in Figure 12.

The mathematical model of the WBD problem is given by Equation (27).

(27) $$\begin{array}{c} Minf\left(x\right)=1.10471x_{1}^{2}x_{2}+0.04811x_{3}x_{4}\times \left(14+x_{2}\right)\\ x=\left[x_{1},x_{2},x_{3},x_{3}\right]=\left[h,l,t,b\right].\\ s.t\left\{\begin{array}{c} f_{1}\left(x\right)=\tau \left(x\right)-\tau_{\max}\leq 0,\\ f_{2}\left(x\right)=\sigma \left(x\right)-\sigma_{\max}\leq 0,\\ f_{3}\left(x\right)=x_{1}-x_{4}\leq 0,\\ f_{4}\left(x\right)=0.125-x_{1}\leq 0,\\ f_{5}\left(x\right)=0.10472x_{1}^{2}+0.04821x_{3}x_{4}\left(14+x_{2}\right)-5\leq 0,\\ f_{5}\left(x\right)=0.125-x_{1}\leq 0,\\ f_{6}\left(x\right)=\delta \left(x\right)-\delta_{\max}\leq 0,\\ f_{7}\left(x\right)=P-P_{c}\left(x\right)\leq 0,\\ \tau \left(x\right)=\sqrt{{\left(\tau^{\prime }\right)}^{2}+2\tau^{\prime }\tau^{\prime \prime }\frac{x_{2}}{R}+\tau^{2}},\tau^{\prime }=\frac{P}{\sqrt{2}x_{1}x_{2}},\tau^{\prime \prime }=\frac{MR}{J},\\ M=P\left(L+\frac{x_{2}}{2}\right),R=\sqrt{\frac{x_{2}^{2}}{4}+{\left(\frac{x_{1}+x_{2}}{2}\right)}^{2}},J=2\left\{\sqrt{2}x_{1}x_{2}\left[\frac{x_{2}^{2}}{12}+{\left(\frac{x_{1}+x_{3}}{2}\right)}^{2}\right]\right\},\\ \sigma \left(x\right)=\frac{6PL}{x_{4}x_{3}^{2}},\delta \left(x\right)=\frac{6PL^{3}}{Ex_{4}x_{3}^{2}},\\ P_{c}\left(x\right)=\frac{4.013E\sqrt{\frac{x_{3}^{2}x_{4}^{6}}{36}}}{L^{2}}\left(1-\frac{x_{3}}{2L}\sqrt{\frac{E}{4G}}\right),\\ \tau_{\max}=13600psi,\sigma_{\max}=30000psi,\delta_{\max}=0.25in,\\ P=60001b,E=30E+10^{6}psi,G=12E+10^{6}psi,L=14. \end{array}\right. \end{array}$$

The statistical results, including the optimal parameters and objective function values obtained by various algorithms, are summarized in Table 12.

It can be observed from Table 12 that the MSECOA significantly outperforms other comparative algorithms in addressing the WBD problem. The best objective function value, mean, and standard deviation achieved by the MSECOA are reported as 1.6928, 1.6928, and 0.0001, respectively, surpassing the performance of all other algorithms. Specifically, the optimal solution derived by the MSECOA is x={0.2057,3.2349,9.0366,0.2057}, yielding a minimum objective function value of 1.6928, which is notably lower than the results of the other methods. In contrast, algorithms such as the ICOA, COA, and WOA exhibit inferior performance, with their best objective function values recorded as 1.9303, 1.8437, and 1.7236, respectively—substantially higher than that of the MSECOA. Additionally, although the LSHADE_cnEpSin algorithm demonstrates performance relatively close to that of the MSECOA, its best objective function value and standard deviation remain less competitive than those achieved by the MSECOA.

### 6.3. Tension/Compression Spring Design

**Figure 13:**
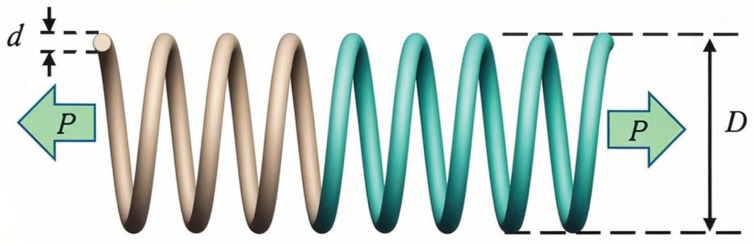
Schematic of tension/compression spring design problem.

The tension/compression spring design (TCSD) problem [40] is a classic case in engineering optimization, aimed at minimizing the weight of a spring by optimizing its geometric parameters—namely, the wire diameter $d(x_{1})$, mean coil diameter $D(x_{2})$, and number of active coils $N(x_{3})$—while adhering to constraints such as minimum deflection $g_{1}$, shear stress $g_{2}$, oscillation frequency $g_{3}$, and outer diameter limit $g_{4}$. A schematic of the TCSD problem is illustrated in Figure 13.

The mathematical model for the TCSD problem is expressed in Equation (28).

(28) $$\begin{array}{c} minf\left(x\right)=\left(2+x_{3}\right)x_{1}^{2}x_{2}\\ x=\left[x_{1},x_{2},x_{3}\right]=\left[d,D,N\right]\\ s.t\left\{\begin{array}{c} g_{1}\left(x\right)=1-\frac{x_{2}^{3}x_{3}}{71785x_{1}^{4}}⩽0\\ g_{2}\left(x\right)=\frac{4x_{2}^{2}-x_{1}x_{2}}{12566\left(\;x_{1}^{3}x_{2}-x_{1}^{4}\right)}+\frac{1}{5108x_{1}^{2}}-1⩽0\\ g_{3}\left(x\right)=1-\frac{140.45x_{1}}{x_{2}^{2}x_{3}}⩽0\\ g_{4}\left(x\right)=\frac{x_{1}x_{2}}{1.5}-1⩽0\\ 0.05⩽x_{1}⩽2,0.25⩽x_{2}⩽1.3,2⩽x_{3}⩽15 \end{array}\right. \end{array}$$

The statistical results, including the optimal parameters and objective function values obtained by various algorithms—such as the best value, mean, standard deviation, and ranking—are presented in Table 13.

Table 13 lists the optimal parameters and statistical results of the objective function for each algorithm in the compression spring design problem. From the table, it is evident that the MSECOA significantly outperforms the other compared algorithms in this problem. Its optimal value, mean, and standard deviation of the objective function are 0.012665, 0.012665, and $7.8035\times 10^{-8}$, respectively, all superior to those of the other algorithms, demonstrating its efficiency and stability in solving complex engineering problems. Specifically, the optimal solution obtained by the MSECOA is x={0.0517,0.3567,11.2892}, corresponding to a minimum objective function value of 0.012665, which is notably lower than the results of the other algorithms.

### 6.4. Pressure Vessel Design

**Figure 14:**
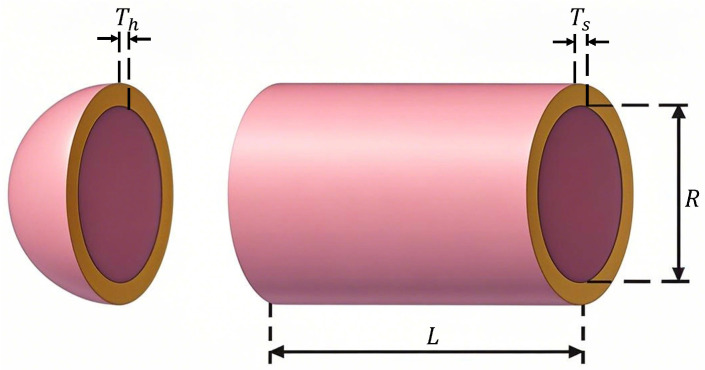
Schematic of pressure vessel design problem.

The pressure vessel design (PVD) problem [41] aims to minimize the manufacturing cost of a pressure vessel by optimizing multiple design variables. These variables include shell thickness $T_{s}$, head thickness $T_{n}$, inner radius *R*, and length *L*, which directly affect the vessel’s cost, safety, and performance. During the optimization process, these variables need to be comprehensively considered to ensure that the cost is minimized while satisfying the engineering constraints. A schematic of the PVD problem is illustrated in in Figure 14.

The mathematical model of the PVD problem is represented as shown in Equation (29).

(29) $$\begin{array}{c} \min f\left(x\right)=0.6224x_{1}x_{3}x_{4}+1.7781x_{2}x_{2}^{2}+3.1661x_{1}^{2}x_{4}+19.84x_{1}^{2}x_{3}\\ x=\left[x_{1},x_{2},x_{3},x_{4}\right]=\left[T_{s},T_{n},R,L\right]\\ s.t\left\{\begin{array}{c} g_{1}=-x_{1}+0.0193x_{3}\leq 0\\ g_{2}=-x_{2}+0.00954x_{3}\leq 0\\ g_{3}=-\pi {x_{3}}^{2}x_{4}+\frac{4}{3}\pi {x_{3}}^{3}+129600\leq 0\\ g_{4}=x_{4}-240\leq 0\\ 0\leq x_{1},x_{2}\leq 100,0\leq x_{3},x_{4}\leq 200 \end{array}\right. \end{array}$$

where $x_{1}$ is shell thickness $T_{s}$, $x_{1}$ is head thickness $T_{n}$, $x_{1}$ is the inner radius *R*, and $x_{4}$ is length *L*.

The optimal parameters and objective function statistics—optimal value, mean, standard deviation, and ranking—for each algorithm are summarized in Table 14.

From Table 14, it can be observed that the MSECOA significantly outperforms the other compared algorithms in the pressure vessel design problem. The optimal value, mean, and standard deviation of its objective function are 5885.3328, 5885.4843, and 0.6666, respectively, all of which surpass those of the other algorithms, demonstrating its efficiency and stability in solving complex engineering problems. Specifically, optimal solution obtained by the MSECOA is x={0.7782,0.3846,40.3196,200}, corresponding to a minimum objective function value of 5885.33277, which is notably lower than results of the other algorithms. Additionally, although the LSHADE_cnEpSin algorithm performs relatively close to the MSECOA, its optimal value and standard deviation remain inferior to those of the MSECOA.

## 7. MSECOA for Said–Ball Curves Degree Reduction

### 7.1. Algorithm Design

#### 7.1.1. Population Initialization

The coati population consists of feasible solutions representing the control vertices of the reduced-degree Said–Ball curve. A population initialization method for Said–Ball curve degree reduction is proposed in this paper, aimed at ensuring the quality and diversity of the initial solutions. Given the n+1 control vertices ${\left\{P_{k}\right\}}_{k=0}^{n}$ of the original Said–Ball curve, their coordinate range is first determined:

(30) $$P_{\min}={\left[\min_{k}x_{k},\min_{k}y_{k}\right]}^{T},P_{\max}={\left[\max_{k}x_{k},\max_{k}y_{k}\right]}^{T}$$

To ensure end point interpolation, $Q_{0}=P_{0}$ and $Q_{m}=P_{n}$ are set. Thus, for the m+1 control vertices ${\left\{Q_{j}\right\}}_{j=0}^{m}$ of the reduced-degree curve, the generation rule is given by Equation (31):

(31) $$Q_{j}=\left\{\begin{array}{cc} P_{0}, & j=0\\ P_{\min}+\alpha_{j}\circ \left(P_{\max}-P_{\min}\right), & j=1,...,m-1\\ P_{n}, & j=m \end{array}\right.$$

where $Q_{j}\in R^{2}$ denotes the coordinate vector of the *j*-th control vertex, $\alpha_{j}∼U\left({\left[0,1\right]}^{2}\right)$ is a two-dimension uniformly distributed random vector, and ∘ represents the Hadamard product (element-wise multiplication).

To ensure population diversity, the search space is expanded, as shown in Equation (32):

(32) $$\begin{array}{cc} \Omega = & \{Q_{j}\in R^{2}|x_{\min}-\rho \Delta x\leq x_{j}\leq x_{\max}+\rho \Delta x,\\ \; & y_{\min}-\rho \Delta y\leq y_{j}\leq y_{\max}+\rho \Delta y\} \end{array}$$

where $\Delta x=x_{\max}-x_{\min}$, $\Delta y=y_{\max}-y_{\min}$, and ρ∈(0,1] is the space expansion factor.

#### 7.1.2. Selection of Fitness Function

In a population, an individual’s fitness is determined by the quality of its properties, with higher fitness values increasing the likelihood of selection for breeding the next generation. To meet the approximation conditions of the Said–Ball curve $P_{n}\left(t\right)$, the objective function is defined as minimizing the Euclidean distance term and average curvature difference between the original curve and reduced-degree curve over the parameter range. Specifically, the Euclidean distance term is used to quantify the geometric deviation between the reduced-degree curve and original curve, ensuring that the reduced-degree curve remains as close as possible to the original in terms of geometric position, thus reducing the overall approximation error. The average curvature difference is employed to assess the ability of the reduced-degree curve to preserve shape characteristics, particularly in curved regions, ensuring smooth transitions and the retention of geometric features while avoiding shape distortion or abrupt changes. By combining the Euclidean distance term with the average curvature difference, the objective function is designed to maintain the curve’s shape properties effectively while ensuring degree reduction accuracy. The mathematical expression of the objective function is given in Equation (33):

(33) $$\mathrm{Minimize}\;F\left(P_{n}\left(t\right),Q_{m}\left(t\right)\right)=\lambda \;\cdot \;\int_{0}^{1}d{\left(P_{n}\left(t\right),Q_{m}\left(t\right)\right)}^{2}dt+\left(1-\lambda \right)\;\cdot \;\int_{0}^{1}{\left(\kappa_{1}\left(t\right)-\kappa_{2}\left(t\right)\right)}^{2}dt.$$

here, $P_{n}\left(t\right)$ and $Q_{m}\left(t\right)$ represent parameterized expressions of the original and reduced-degree curves, respectively; $d(P_{n}\left(t\right),Q_{m}\left(t\right))$ denotes the Euclidean distance between the original and reduced-degree curves over the parameter range; $\kappa_{1}\left(t\right)$ and $\kappa_{2}\left(t\right)$ indicate the curvatures of the original and reduced-degree curves at parameter *t*, respectively; and λ is a weighting coefficient used to balance the Euclidean distance term and the average curvature difference, set to 0.8.

By appropriately setting the weighting coefficient λ, a dynamic balance between geometric accuracy and shape preservation can be achieved during the degree reduction process, thereby providing a more effective solution for the degree reduction of complex curves.

#### 7.1.3. Degree Reduction Error

To evaluate the error between curves before and after degree reduction, an integral-based error calculation formula is adopted in this paper, as shown in Equation (34):

(34) $$\varepsilon =D\left(P_{n}\left(t\right)-Q_{m}\left(t\right)\right)=\int_{0}^{1}{\left(P_{n}\left(t\right)-Q_{m}\left(t\right)\right)}^{2}\;dt.$$

here, $P_{n}\left(t\right)$ represents the control vertex function of the original curve, $Q_{m}\left(t\right)$ denotes the control vertex function of the reduced-degree curve, and ε is the degree reduction error, used to quantify the difference between the curves before and after reduction. This formula, by calculating the integral of the squared difference between the two curves over the parameter interval [0,1], comprehensively reflects the deviation in the overall shape of the curves before and after degree reduction. A smaller degree reduction error ε indicates a higher degree of fit between the reduced curve and original curve, signifying a better degree reduction outcome.

### 7.2. Algorithm Steps

Based on introduction to the MSECOA in Section 4, the COA iteratively updates its position in the search space through exploration and exploitation phases to find the optimal solution. Therefore, in the degree reduction process of the Said–Ball curves, the curve’s control vertices are treated as coati’s position information, and control vertices satisfying the degree reduction condition are continuously updated through the position update strategy to obtain the reduced-degree Said–Ball curve.

The specific implementation steps for applying the MSECOA to Said–Ball curve degree reduction are as follows:

**Step 1:** Initialize the control vertex sequence of the Said–Ball curve $p_{0},p_{1},\ldots ,p_{n}$, and the set target reduction degree as *m*.

**Step 2**: Set the population size *N* and maximum number of iterations *T*, and initialize the iteration counter t=0.

**Step 3**: Generate the initial coati population $X_{i}$ according to Equations (30)–(32), where (i=1,2,…,N).

**Step 4**: Calculate the fitness value $f(X_{i})$ for each coati individual.

**Step 5**: Update the positions of the coati individuals based on the position update strategies of the exploration and exploitation phases.

**Step 6**: Update the current best solution $X_{\mathrm{best}}$ and its corresponding fitness value $f_{\mathrm{best}}$.

**Step 7**: If the current iteration count *t* reaches the maximum *T*, proceed to Step 8; otherwise, increment t=t+1 and return to Step 4.

**Step 8**: Output the optimal solution $X_{\mathrm{best}}$ and its corresponding sequence of reduced-degree control vertices.

**Step 9**: Plot the reduced-degree Said–Ball curve and compute the degree reduction error.

### 7.3. Numerical Examples

To verify the performance of the MSECOA in Said–Ball curve degree reduction, this paper selects multiple cases with different degree reduction levels for testing. In the experiment, the MSECOA is compared with four algorithms: the basic COA, the improved coati optimization algorithm (ICOA) [30], the whale optimization algorithm (WOA) [26], and the hiking optimization algorithm (HOA) [35]). Additionally, the population size is uniformly set to 30 and the maximum number of iterations to 200 for all algorithms to ensure fairness in comparison. Each algorithm is independently run 30 times to mitigate the impact of randomness on results, and the mean, standard deviation, and minimum values of the degree reduction error are calculated as performance evaluation metrics.

#### 7.3.1. Example 1

The control points for example 1 are defined as follows:

(35) $$\begin{array}{cc} \; & \{P_{0}=\left(0.1,0\right),P_{1}=\left(0,0.1\right),P_{2}=\left(0.05,0.2\right),P_{3}=\left(0.3,0.3\right),\\ \; & P_{4}=\left(0.55,0.2\right),P_{5}=\left(0.6,0.1\right),P_{6}=\left(0.5,0\right)\} \end{array}$$

An sixth-degree Said–Ball curve was constructed using these control points and was subsequently reduced to a fourth-degree curve through various algorithms, without employing end point interpolation constraints. The resulting control point coordinates and statistical error metrics for each algorithm are presented in Table 15.

From Table 15, it can be observed that the MSECOA performs best in Case 1, with the mean, standard deviation, and minimum values of its degree reduction error (ε) significantly lower than those of the other algorithms. Specifically, the MSECOA achieves a mean error of $3.671\times 10^{-5}$, a standard deviation of $2.710\times 10^{-6}$, and a minimum error of $3.351\times 10^{-5}$, all of which outperform the COA, ICOA, WOA, and HOA algorithms. This indicates that the MSECOA exhibits higher convergence accuracy and stability during the degree reduction process. Furthermore, the reduced-degree curve generated by the MSECOA approximates the original curve more closely, whereas the other algorithms tend to get trapped in local optima during degree reduction, resulting in larger errors. For instance, the mean errors of the COA and ICOA are $2.180\times 10^{-3}$ and $3.149\times 10^{-3}$, respectively, notably higher than that of the MSECOA.

**Figure 15:**
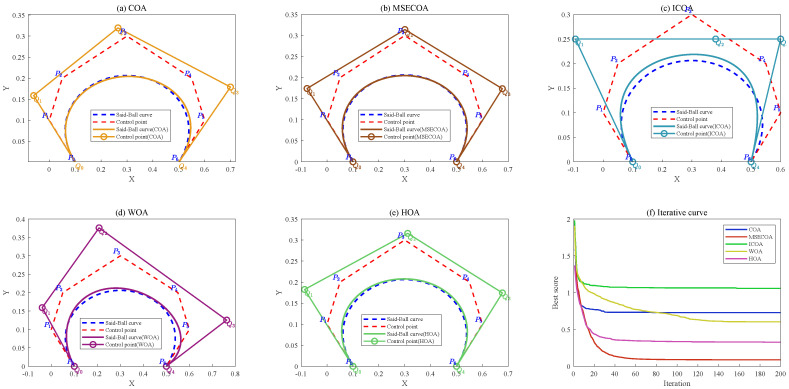
Degree reduction results of different algorithms (example 1).

Figure 15 illustrates the comparative results of reducing a seventh-degree Said–Ball curve to a fourth-degree representation, including both the geometric approximation quality and iterative convergence behavior.

#### 7.3.2. Example 2

The control points for example 2 are defined as follows:

(36) $$\begin{array}{cc} \; & \{P_{0}=\left(-0.871,0.408\right),P_{1}=\left(-0.692,0.916\right),P_{2}=\left(-0.483,0.597\right),P_{3}=\left(-0.256,0.302\right),\\ \; & P_{4}=\left(0.256,0.302\right),P_{5}=\left(0.302;0.483\right),P_{6}=\left(0.692,0.916\right),P_{7}=\left(0.871,0.409\right)\} \end{array}$$

An seventh-degree Said–Ball curve was constructed using these control points and was subsequently reduced to a fourth-degree curve through various algorithms, without employing end point interpolation constraints. The resulting control point coordinates and statistical error metrics for each algorithm are presented in Table 16.

The statistical results presented in Table 16 demonstrate that the MSECOA achieves significantly lower degree-reduction errors (ε) compared to alternative methods. Specifically, the MSECOA exhibits a mean error of $5.704\times 10^{-4}$ with a standard deviation of $4.420\times 10^{-6}$ and minimum error of $5.628\times 10^{-4}$, outperforming the COA, ICOA, WOA, and HOA. In contrast, the COA and ICOA show substantially higher mean errors of $1.295\times 10^{-2}$ and $4.988\times 10^{-3}$, respectively. The algorithm’s superior performance in both standard deviation and minimum error metrics further confirms its enhanced stability and convergence precision during the degree reduction process.

**Figure 16:**
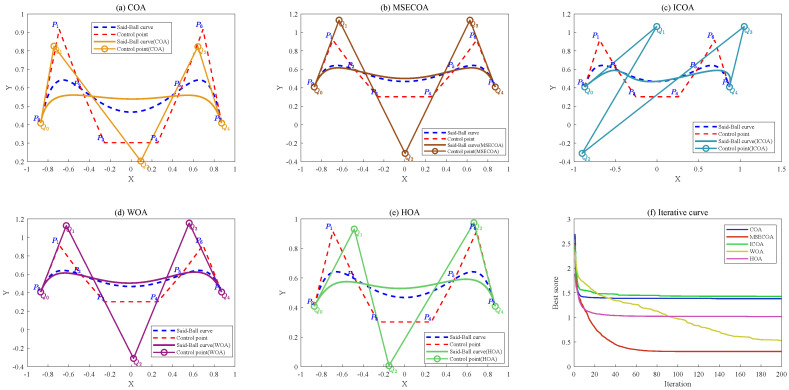
Degree reduction results of different algorithms (example 2).

Figure 16 illustrates comparative results of reducing a seventh-degree Said–Ball curve to a fourth-degree representation, including both the geometric approximation quality and iterative convergence behavior.

#### 7.3.3. Example 3

The control points for example 3 are defined as follows:

(37) $$\begin{array}{cc} \; & \{P_{0}=\left(-0.5,0\right),P_{1}=\left(-0.8,0.3\right),P_{2}=\left(-0.65,0.7\right),P_{3}=\left(-0.1,1\right),P_{4}=\left(0.6,1.1\right),\\ \; & P_{5}=\left(1.3,1\right),P_{6}=\left(1.85,0.7\right),P_{7}=\left(2,0.3\right),P_{8}=\left(1.7,0\right)\} \end{array}$$

An eighth-degree Said–Ball curve was constructed using these control points and was subsequently reduced to a fourth-degree curve through various algorithms, without employing end point interpolation constraints. The resulting control point coordinates and statistical error metrics for each algorithm are presented in Table 17.

Table 17 demonstrates that the MSECOA achieves significantly lower approximation errors in degree reduction compared to alternative methods. Specifically, the MSECOA exhibits a mean error of $1.059\times 10^{-5}$, standard deviation of $1.070\times 10^{-5}$, and minimum error of $6.720\times 10^{-6}$, outperforming the COA, ICOA, WOA, and HOA by two orders of magnitude. In contrast, the COA and ICOA show substantially higher mean errors of $3.368\times 10^{-2}$ and $4.699\times 10^{-3}$, respectively. The algorithm’s superior performance in both standard deviation and minimum error further confirms its exceptional stability and precision.

**Figure 17:**
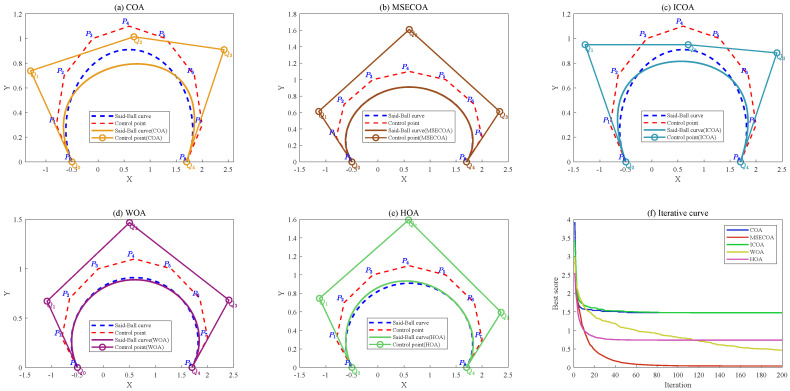
Degree reduction results of different algorithms (example 3).

Figure 17 illustrates the degree reduction comparison from eighth-order to fourth-order Said–Ball curves, displaying both the geometric approximation and convergence behavior.

#### 7.3.4. Example 4

The control points for example 4 are defined as follows:

(38) $$\begin{array}{cc} \; & \{P_{0}=\left(-0.3,0\right),P_{1}=\left(-0.8,0.3\right),P_{2}=\left(-0.65,0.7\right),P_{3}=\left(-0.1,1.0\right),P_{4}=\left(0.6,1.1\right),\\ \; & P_{5}=\left(1.3,1.0\right),P_{6}=\left(1.85,0.7\right),P_{7}=\left(2.0,0.3\right),P_{8}=\left(1.5,0\right)\} \end{array}$$

An eighth-degree Said–Ball curve was constructed from these control points and subsequently reduced to a third-degree curve using different algorithms without end point interpolation constraints. The resulting control point coordinates and statistical error metrics for each method are presented in Table 18.

As evidenced by the statistical results in Table 18, the MSECOA demonstrates significantly lower degree-reduction errors compared to the other methods. Specifically, the MSECOA achieves a mean error of $1.353\times 10^{-3}$ with a standard deviation of $8.900\times 10^{-7}$, while the minimum error ($1.350\times 10^{-3}$) closely aligns with the mean value. This consistency confirms the algorithm’s stability across varying initial conditions. In contrast, the COA and ICOA exhibit notably higher mean errors ($1.844\times 10^{-2}$ and $2.567\times 10^{-3}$, respectively). Furthermore, the MSECOA’s superior performance in both the standard deviation and minimum error metrics underscores its enhanced stability and convergence precision during degree reduction. These results collectively demonstrate that the MSECOA offers a robust solution for Said–Ball curve degree reduction, effectively minimizing approximation errors while preserving geometric characteristics. The algorithm thus presents an efficient and reliable approach for handling complex curve simplification.

**Figure 18:**
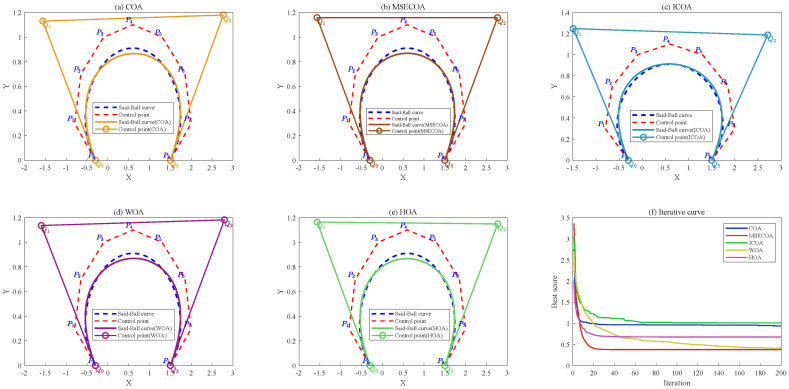
Degree reduction results of different algorithms (example 4).

Figure 18 illustrates the comparative results of reducing an eighth-degree Said–Ball curve to a third-degree curve, including both the curve geometry and iterative convergence behavior.

## 8. Conclusions and Future Prospects

This paper addresses the degree reduction problem of Said–Ball curves by proposing a multi-strategy enhanced coati optimization algorithm (MSECOA). The algorithm constructs a degree reduction model based on Euclidean distance and integrated curvature information, incorporating multiple strategies such as good point set-based hybrid opposition learning, fitness–distance balance, dynamic spiral searching, and adaptive differential evolution. These enhancements significantly improve the algorithm’s global search capability, local exploitation ability, and convergence speed. The experimental results demonstrate that the MSECOA outperforms the existing methods on the IEEE CEC2017 and CEC2022 test function suites and exhibits strong practical applicability in four engineering constrained design problems. Furthermore, numerical experiments with four different degree reduction levels validate the MSECOA’s notable advantages in the Said–Ball curve degree reduction problem, effectively preserving the geometric features of the curve while reducing its degree. This provides an efficient and reliable solution for complex curve degree reduction challenges.

The potential applications of the MSECOA extend beyond the degree reduction of Said–Ball curves to broader areas of geometric modeling [42,43]. In particular, the MSECOA demonstrates strong applicability in curve and surface modeling for complex real-world problems. For example, in vascular structure reconstruction, accurate curve approximation is essential for modeling intricate blood vessel geometries based on segmented medical imaging data. The algorithm’s ability to preserve shape characteristics during degree reduction makes it particularly suitable for reconstructing tubular anatomical structures, such as arteries and veins, under spatial constraints. Similarly, in pipeline design, where the precise geometric representation of pipe networks is crucial for layout optimization and structural integrity analysis, the MSECOA can be applied to generate high-fidelity free-form curves and surfaces, offering improved efficiency and accuracy.

Although the MSECOA has demonstrated notable performance advantages in current degree reduction tasks, future research will focus on further improving its convergence behavior and robustness. Promising research directions include extending the algorithm to handle curves defined over disk or ball domains, exploring its application in the degree reduction of Ball Said–Ball curves, and generalizing the framework to high-dimensional surface reduction, thereby addressing more complex geometric modeling challenges. Additionally, integrating reinforcement learning techniques to explore the MSECOA’s potential in intelligent geometric modeling represents a promising avenue for future studies.

## Figures and Tables

**Table 1:** Algorithm parameter settings.

Algorithm	Parameter Settings
MSECOA	CR∈[0.1,0.9],I∈[1,2]
COA	I∈[1,2]
ICOA	v=0.3,a∈[1,2]
WOA	a∈[2,0],b=1,l∈[−1,1]
AOA	${\mathrm{MOP}}_{\mathrm{Max}}=1,{\mathrm{MOP}}_{\mathrm{Min}}=0.2,A=5,Mu=0.499$
NCHHO	$c\in \left[2,0\right],a=4,X_{n}=0.7$
ISCSO	C=0.01,β=2,δ=2,SM=2
BKA	P=0.9
HOA	$Sweep\;Factor\;f\in \left[2,0\right],Angle\;of\;inclination\in \left[0,50^{\circ }\right]$
LSHADE_cnEpSin	$\begin{array}{cc} NP_{min} & =4.0,Pbest_{rate}=0.11,Arc_{rate}=1.4,\\ & H=5,\mu_{Finitial}=\mu_{CRinitial}=0.5 \end{array}$

**Table 2:** IEEE CEC2017 benchmark functions.

Type	Function No	Function Description	Range	Optimum
Unimodal	F1	Shifted and rotated bent cigar function	[−100, 100]	100
Unimodal	F3	Shifted and rotated Zakharov function	[−100, 100]	300
Multimodal	F4	Shifted and rotated Rosenbrock’s function	[−100, 100]	400
Multimodal	F5	Shifted and rotated Rastrigin’s function	[−100, 100]	500
Multimodal	F6	Shifted and rotated expanded Scaffer’s F6 function	[−100, 100]	600
Multimodal	F7	Shifted and rotated Lunacek Bi-Rastrigin function	[−100, 100]	700
Multimodal	F8	Shifted and rotated Non-continuous Rastrigin’s function	[−100, 100]	800
Multimodal	F9	Shifted and rotated Levy function	[−100, 100]	900
Multimodal	F10	Shifted and rotated Schwefel’s function	[−100, 100]	1000
Hybrid	F11	Hybrid function 1 (*N* = 3)	[−100, 100]	1100
Hybrid	F12	Hybrid function 2 (*N* = 3)	[−100, 100]	1200
Hybrid	F13	Hybrid function 3 (*N* = 3)	[−100, 100]	1300
Hybrid	F14	Hybrid function 4 (*N* = 4)	[−100, 100]	1400
Hybrid	F15	Hybrid function 4 (*N* = 4)	[−100, 100]	1500
Hybrid	F16	Hybrid function 6 (*N* = 4)	[−100, 100]	1600
Hybrid	F17	Hybrid function 6 (*N* = 5)	[−100, 100]	1700
Hybrid	F18	Hybrid function 6 (*N* = 5)	[−100, 100]	1800
Hybrid	F19	Hybrid function 6 (*N* = 5)	[−100, 100]	1900
Hybrid	F20	Hybrid function 6 (*N* = 5)	[−100, 100]	2000
Composition	F21	Composition function 1 (*N* = 3)	[−100, 100]	2100
Composition	F22	Composition function 2 (*N* = 3)	[−100, 100]	2200
Composition	F23	Composition function 3 (*N* = 4)	[−100, 100]	2300
Composition	F24	Composition function 4 (*N* = 4)	[−100, 100]	2400
Composition	F25	Composition function 5 (*N* = 5)	[−100, 100]	2500
Composition	F26	Composition function 6 (*N* = 5)	[−100, 100]	2600
Composition	F27	Composition function 7 (*N* = 6)	[−100, 100]	2700
Composition	F28	Composition function 8 (*N* = 6)	[−100, 100]	2800
Composition	F29	Composition function 9 (*N* = 3)	[−100, 100]	2900
Composition	F30	Composition function 10 (*N* = 3)	[−100, 100]	3000

**Table 3:** Results of CEC2017 in dimension 10.

Fun	Index	MSECOA	ICOA	COA	WOA	AOA	BKA	HOA	NCHHO	ISCSO	LSHADE_cnEpSin
F1	Mean	$1.968\times 10^{5}$	$8.878\times 10^{9}$	$9.361\times 10^{9}$	$8.268\times 10^{6}$	$7.114\times 10^{9}$	$1.101\times 10^{8}$	$4.297\times 10^{9}$	$7.411\times 10^{9}$	$1.524\times 10^{10}$	$2.022\times 10^{6}$
	Std	$6.941\times 10^{5}$	$2.403\times 10^{9}$	$2.717\times 10^{9}$	$1.742\times 10^{7}$	$2.961\times 10^{9}$	$3.517\times 10^{8}$	$2.220\times 10^{9}$	$2.861\times 10^{9}$	$6.940\times 10^{9}$	$1.129\times 10^{6}$
F3	Mean	$3.019\times 10^{2}$	$8.222\times 10^{3}$	$9.933\times 10^{3}$	$5.280\times 10^{3}$	$1.050\times 10^{4}$	$1.246\times 10^{3}$	$8.776\times 10^{3}$	$9.441\times 10^{3}$	$1.777\times 10^{4}$	$3.272\times 10^{3}$
	Std	$6.008\times 10^{0}$	$1.989\times 10^{3}$	$2.448\times 10^{3}$	$4.220\times 10^{3}$	$2.628\times 10^{3}$	$3.027\times 10^{3}$	$3.002\times 10^{3}$	$3.670\times 10^{3}$	$4.497\times 10^{3}$	$1.484\times 10^{3}$
F4	Mean	$4.029\times 10^{2}$	$1.012\times 10^{3}$	$1.133\times 10^{3}$	$4.307\times 10^{2}$	$1.099\times 10^{3}$	$4.477\times 10^{2}$	$6.111\times 10^{2}$	$8.501\times 10^{2}$	$1.876\times 10^{3}$	$4.072\times 10^{2}$
	Std	$1.485\times 10^{0}$	$1.879\times 10^{2}$	$3.040\times 10^{2}$	$3.558\times 10^{1}$	$4.659\times 10^{2}$	$1.263\times 10^{2}$	$1.297\times 10^{2}$	$3.259\times 10^{2}$	$1.088\times 10^{3}$	$7.313\times 10^{-1}$
F5	Mean	$5.122\times 10^{2}$	$5.941\times 10^{2}$	$5.906\times 10^{2}$	$5.594\times 10^{2}$	$5.535\times 10^{2}$	$5.368\times 10^{2}$	$5.543\times 10^{2}$	$5.814\times 10^{2}$	$6.162\times 10^{2}$	$5.257\times 10^{2}$
	Std	$4.186\times 10^{0}$	$1.238\times 10^{1}$	$1.699\times 10^{1}$	$2.039\times 10^{1}$	$2.092\times 10^{1}$	$1.417\times 10^{1}$	$1.485\times 10^{1}$	$1.878\times 10^{1}$	$2.877\times 10^{1}$	$5.329\times 10^{0}$
F6	Mean	$6.003\times 10^{2}$	$6.429\times 10^{2}$	$6.480\times 10^{2}$	$6.375\times 10^{2}$	$6.404\times 10^{2}$	$6.268\times 10^{2}$	$6.286\times 10^{2}$	$6.433\times 10^{2}$	$6.671\times 10^{2}$	$6.011\times 10^{2}$
	Std	$6.515\times 10^{-1}$	$7.558\times 10^{0}$	$9.222\times 10^{0}$	$1.431\times 10^{1}$	$8.403\times 10^{0}$	$1.072\times 10^{1}$	$9.214\times 10^{0}$	$1.323\times 10^{1}$	$1.026\times 10^{1}$	$4.119\times 10^{-1}$
F7	Mean	$7.313\times 10^{2}$	$7.951\times 10^{2}$	$8.038\times 10^{2}$	$7.862\times 10^{2}$	$7.974\times 10^{2}$	$7.548\times 10^{2}$	$7.638\times 10^{2}$	$8.087\times 10^{2}$	$8.407\times 10^{2}$	$7.403\times 10^{2}$
	Std	$1.452\times 10^{1}$	$1.525\times 10^{1}$	$1.900\times 10^{1}$	$2.304\times 10^{1}$	$1.567\times 10^{1}$	$1.905\times 10^{1}$	$1.411\times 10^{1}$	$1.999\times 10^{1}$	$2.490\times 10^{1}$	$6.116\times 10^{0}$
F8	Mean	$8.145\times 10^{2}$	$8.560\times 10^{2}$	$8.567\times 10^{2}$	$8.427\times 10^{2}$	$8.317\times 10^{2}$	$8.226\times 10^{2}$	$8.339\times 10^{2}$	$8.461\times 10^{2}$	$8.732\times 10^{2}$	$8.247\times 10^{2}$
	Std	$4.886\times 10^{0}$	$5.351\times 10^{0}$	$9.471\times 10^{0}$	$1.386\times 10^{1}$	$5.884\times 10^{0}$	$7.991\times 10^{0}$	$6.346\times 10^{0}$	$8.798\times 10^{0}$	$1.063\times 10^{1}$	$5.971\times 10^{0}$
F9	Mean	$9.273\times 10^{2}$	$1.478\times 10^{3}$	$1.537\times 10^{3}$	$1.407\times 10^{3}$	$1.382\times 10^{3}$	$1.188\times 10^{3}$	$1.155\times 10^{3}$	$1.596\times 10^{3}$	$2.325\times 10^{3}$	$9.034\times 10^{2}$
	Std	$4.812\times 10^{1}$	$1.859\times 10^{2}$	$2.063\times 10^{2}$	$3.356\times 10^{2}$	$1.781\times 10^{2}$	$1.545\times 10^{2}$	$1.261\times 10^{2}$	$2.369\times 10^{2}$	$3.892\times 10^{2}$	$2.306\times 10^{0}$
F10	Mean	$1.427\times 10^{3}$	$2.587\times 10^{3}$	$2.538\times 10^{3}$	$2.074\times 10^{3}$	$2.182\times 10^{3}$	$1.830\times 10^{3}$	$2.274\times 10^{3}$	$2.461\times 10^{3}$	$2.791\times 10^{3}$	$1.972\times 10^{3}$
	Std	$2.176\times 10^{2}$	$1.650\times 10^{2}$	$2.000\times 10^{2}$	$3.589\times 10^{2}$	$2.291\times 10^{2}$	$3.566\times 10^{2}$	$2.861\times 10^{2}$	$3.264\times 10^{2}$	$4.810\times 10^{2}$	$2.056\times 10^{2}$
F11	Mean	$1.113\times 10^{3}$	$2.161\times 10^{3}$	$2.717\times 10^{3}$	$1.227\times 10^{3}$	$2.561\times 10^{3}$	$1.187\times 10^{3}$	$2.268\times 10^{3}$	$2.788\times 10^{3}$	$1.503\times 10^{4}$	$1.114\times 10^{3}$
	Std	$2.035\times 10^{1}$	$8.538\times 10^{2}$	$1.608\times 10^{3}$	$8.430\times 10^{1}$	$2.499\times 10^{3}$	$1.587\times 10^{2}$	$8.756\times 10^{2}$	$2.134\times 10^{3}$	$1.819\times 10^{4}$	$4.010\times 10^{0}$
F12	Mean	$2.456\times 10^{4}$	$1.717\times 10^{8}$	$2.659\times 10^{8}$	$4.546\times 10^{6}$	$9.242\times 10^{7}$	$2.509\times 10^{5}$	$7.794\times 10^{6}$	$5.298\times 10^{7}$	$7.561\times 10^{8}$	$7.168\times 10^{5}$
	Std	$6.057\times 10^{4}$	$1.467\times 10^{8}$	$2.178\times 10^{8}$	$5.142\times 10^{6}$	$1.334\times 10^{8}$	$1.020\times 10^{6}$	$6.991\times 10^{6}$	$1.415\times 10^{8}$	$5.882\times 10^{8}$	$4.451\times 10^{5}$
F13	Mean	$1.336\times 10^{3}$	$6.829\times 10^{5}$	$8.912\times 10^{5}$	$1.981\times 10^{4}$	$1.097\times 10^{4}$	$1.951\times 10^{3}$	$5.002\times 10^{5}$	$1.619\times 10^{4}$	$8.460\times 10^{7}$	$1.450\times 10^{3}$
	Std	$6.438\times 10^{1}$	$8.620\times 10^{5}$	$3.164\times 10^{6}$	$1.403\times 10^{4}$	$8.205\times 10^{3}$	$4.352\times 10^{2}$	$1.665\times 10^{6}$	$1.232\times 10^{4}$	$1.239\times 10^{8}$	$1.184\times 10^{2}$
F14	Mean	$1.406\times 10^{3}$	$1.593\times 10^{3}$	$1.545\times 10^{3}$	$2.173\times 10^{3}$	$8.826\times 10^{3}$	$1.475\times 10^{3}$	$4.955\times 10^{3}$	$3.060\times 10^{3}$	$6.278\times 10^{3}$	$1.432\times 10^{3}$
	Std	$6.265\times 10^{0}$	$6.825\times 10^{1}$	$4.435\times 10^{1}$	$1.118\times 10^{3}$	$7.941\times 10^{3}$	$2.862\times 10^{1}$	$3.964\times 10^{3}$	$1.520\times 10^{3}$	$1.670\times 10^{4}$	$4.408\times 10^{0}$
F15	Mean	$1.506\times 10^{3}$	$7.526\times 10^{3}$	$8.058\times 10^{3}$	$8.923\times 10^{3}$	$1.648\times 10^{4}$	$1.613\times 10^{3}$	$8.692\times 10^{3}$	$1.092\times 10^{4}$	$7.153\times 10^{4}$	$1.518\times 10^{3}$
	Std	$6.806\times 10^{0}$	$3.171\times 10^{3}$	$3.885\times 10^{3}$	$5.963\times 10^{3}$	$5.107\times 10^{3}$	$8.434\times 10^{1}$	$4.426\times 10^{3}$	$3.889\times 10^{3}$	$1.863\times 10^{5}$	$5.301\times 10^{0}$
F16	Mean	$1.764\times 10^{3}$	$2.107\times 10^{3}$	$2.089\times 10^{3}$	$1.906\times 10^{3}$	$2.038\times 10^{3}$	$1.769\times 10^{3}$	$1.941\times 10^{3}$	$2.025\times 10^{3}$	$2.403\times 10^{3}$	$1.625\times 10^{3}$
	Std	$1.237\times 10^{2}$	$1.087\times 10^{2}$	$1.230\times 10^{2}$	$1.176\times 10^{2}$	$1.373\times 10^{2}$	$1.012\times 10^{2}$	$1.169\times 10^{2}$	$1.674\times 10^{2}$	$2.331\times 10^{2}$	$1.155\times 10^{1}$
F17	Mean	$1.716\times 10^{3}$	$1.786\times 10^{3}$	$1.811\times 10^{3}$	$1.819\times 10^{3}$	$1.880\times 10^{3}$	$1.770\times 10^{3}$	$1.797\times 10^{3}$	$1.802\times 10^{3}$	$1.955\times 10^{3}$	$1.737\times 10^{3}$
	Std	$1.348\times 10^{1}$	$1.945\times 10^{1}$	$3.518\times 10^{1}$	$6.331\times 10^{1}$	$9.836\times 10^{1}$	$3.271\times 10^{1}$	$4.241\times 10^{1}$	$3.168\times 10^{1}$	$6.220\times 10^{1}$	$7.512\times 10^{0}$
F18	Mean	$1.817\times 10^{3}$	$1.187\times 10^{7}$	$3.231\times 10^{7}$	$1.870\times 10^{4}$	$1.570\times 10^{4}$	$2.630\times 10^{3}$	$1.220\times 10^{7}$	$1.524\times 10^{4}$	$1.655\times 10^{8}$	$2.375\times 10^{3}$
	Std	$2.325\times 10^{1}$	$2.509\times 10^{7}$	$8.558\times 10^{7}$	$1.222\times 10^{4}$	$1.032\times 10^{4}$	$1.988\times 10^{3}$	$2.844\times 10^{7}$	$1.259\times 10^{4}$	$3.023\times 10^{8}$	$6.398\times 10^{2}$
F19	Mean	$1.902\times 10^{3}$	$1.283\times 10^{4}$	$1.647\times 10^{4}$	$2.952\times 10^{4}$	$4.212\times 10^{4}$	$1.957\times 10^{3}$	$2.768\times 10^{4}$	$3.389\times 10^{4}$	$6.720\times 10^{6}$	$1.908\times 10^{3}$
	Std	$1.940\times 10^{0}$	$1.970\times 10^{4}$	$2.828\times 10^{4}$	$5.918\times 10^{4}$	$3.737\times 10^{4}$	$4.401\times 10^{1}$	$3.851\times 10^{4}$	$6.544\times 10^{4}$	$2.805\times 10^{7}$	$3.069\times 10^{0}$
F20	Mean	$2.006\times 10^{3}$	$2.223\times 10^{3}$	$2.258\times 10^{3}$	$2.182\times 10^{3}$	$2.158\times 10^{3}$	$2.087\times 10^{3}$	$2.145\times 10^{3}$	$2.236\times 10^{3}$	$2.362\times 10^{3}$	$2.029\times 10^{3}$
	Std	$1.215\times 10^{1}$	$5.191\times 10^{1}$	$3.929\times 10^{1}$	$6.824\times 10^{1}$	$6.381\times 10^{1}$	$5.188\times 10^{1}$	$7.704\times 10^{1}$	$7.323\times 10^{1}$	$1.205\times 10^{2}$	$6.194\times 10^{0}$
F21	Mean	$2.247\times 10^{3}$	$2.346\times 10^{3}$	$2.370\times 10^{3}$	$2.312\times 10^{3}$	$2.325\times 10^{3}$	$2.278\times 10^{3}$	$2.330\times 10^{3}$	$2.339\times 10^{3}$	$2.389\times 10^{3}$	$2.261\times 10^{3}$
	Std	$5.534\times 10^{1}$	$4.579\times 10^{1}$	$4.831\times 10^{1}$	$6.257\times 10^{1}$	$4.054\times 10^{1}$	$6.355\times 10^{1}$	$4.879\times 10^{1}$	$5.368\times 10^{1}$	$5.162\times 10^{1}$	$5.724\times 10^{1}$
F22	Mean	$2.289\times 10^{3}$	$2.926\times 10^{3}$	$3.097\times 10^{3}$	$2.503\times 10^{3}$	$3.041\times 10^{3}$	$2.307\times 10^{3}$	$2.655\times 10^{3}$	$2.739\times 10^{3}$	$3.188\times 10^{3}$	$2.308\times 10^{3}$
	Std	$3.032\times 10^{1}$	$2.329\times 10^{2}$	$3.254\times 10^{2}$	$4.874\times 10^{2}$	$3.168\times 10^{2}$	$3.512\times 10^{1}$	$3.368\times 10^{2}$	$3.443\times 10^{2}$	$4.493\times 10^{2}$	$1.458\times 10^{0}$
F23	Mean	$2.621\times 10^{3}$	$2.709\times 10^{3}$	$2.707\times 10^{3}$	$2.653\times 10^{3}$	$2.724\times 10^{3}$	$2.637\times 10^{3}$	$2.729\times 10^{3}$	$2.688\times 10^{3}$	$2.772\times 10^{3}$	$2.623\times 10^{3}$
	Std	$8.280\times 10^{0}$	$2.651\times 10^{1}$	$3.299\times 10^{1}$	$2.646\times 10^{1}$	$3.403\times 10^{1}$	$2.925\times 10^{1}$	$3.972\times 10^{1}$	$3.549\times 10^{1}$	$3.337\times 10^{1}$	$4.106\times 10^{0}$
F24	Mean	$2.512\times 10^{3}$	$2.819\times 10^{3}$	$2.870\times 10^{3}$	$2.778\times 10^{3}$	$2.818\times 10^{3}$	$2.753\times 10^{3}$	$2.824\times 10^{3}$	$2.814\times 10^{3}$	$2.919\times 10^{3}$	$2.737\times 10^{3}$
	Std	$4.957\times 10^{1}$	$6.486\times 10^{1}$	$8.071\times 10^{1}$	$4.740\times 10^{1}$	$8.994\times 10^{1}$	$7.248\times 10^{1}$	$1.048\times 10^{2}$	$7.236\times 10^{1}$	$4.810\times 10^{1}$	$5.320\times 10^{1}$
F25	Mean	$2.922\times 10^{3}$	$3.363\times 10^{3}$	$3.459\times 10^{3}$	$2.958\times 10^{3}$	$3.283\times 10^{3}$	$2.933\times 10^{3}$	$3.119\times 10^{3}$	$3.182\times 10^{3}$	$3.741\times 10^{3}$	$2.928\times 10^{3}$
	Std	$2.393\times 10^{1}$	$1.402\times 10^{2}$	$2.002\times 10^{2}$	$2.996\times 10^{1}$	$2.201\times 10^{2}$	$4.095\times 10^{1}$	$1.261\times 10^{2}$	$1.405\times 10^{2}$	$3.539\times 10^{2}$	$2.113\times 10^{1}$
F26	Mean	$2.913\times 10^{3}$	$4.034\times 10^{3}$	$4.083\times 10^{3}$	$3.598\times 10^{3}$	$3.887\times 10^{3}$	$3.135\times 10^{3}$	$3.775\times 10^{3}$	$4.028\times 10^{3}$	$4.591\times 10^{3}$	$2.977\times 10^{3}$
	Std	$6.216\times 10^{1}$	$3.566\times 10^{2}$	$4.017\times 10^{2}$	$5.705\times 10^{2}$	$3.420\times 10^{2}$	$2.868\times 10^{2}$	$3.764\times 10^{2}$	$4.031\times 10^{2}$	$4.490\times 10^{2}$	$2.340\times 10^{1}$
F27	Mean	$3.095\times 10^{3}$	$3.154\times 10^{3}$	$3.196\times 10^{3}$	$3.158\times 10^{3}$	$3.249\times 10^{3}$	$3.108\times 10^{3}$	$3.242\times 10^{3}$	$3.190\times 10^{3}$	$3.322\times 10^{3}$	$3.091\times 10^{3}$
	Std	$3.243\times 10^{0}$	$2.466\times 10^{1}$	$4.077\times 10^{1}$	$5.317\times 10^{1}$	$7.000\times 10^{1}$	$2.799\times 10^{1}$	$4.913\times 10^{1}$	$4.531\times 10^{1}$	$1.054\times 10^{2}$	$9.790\times 10^{-1}$
F28	Mean	$3.295\times 10^{3}$	$3.740\times 10^{3}$	$3.758\times 10^{3}$	$3.422\times 10^{3}$	$3.723\times 10^{3}$	$3.265\times 10^{3}$	$3.701\times 10^{3}$	$3.610\times 10^{3}$	$3.861\times 10^{3}$	$3.226\times 10^{3}$
	Std	$1.435\times 10^{2}$	$1.102\times 10^{2}$	$8.844\times 10^{1}$	$1.431\times 10^{2}$	$1.467\times 10^{2}$	$1.540\times 10^{2}$	$8.808\times 10^{1}$	$1.938\times 10^{2}$	$9.509\times 10^{1}$	$4.584\times 10^{1}$
F29	Mean	$3.185\times 10^{3}$	$3.424\times 10^{3}$	$3.433\times 10^{3}$	$3.338\times 10^{3}$	$3.425\times 10^{3}$	$3.256\times 10^{3}$	$3.302\times 10^{3}$	$3.402\times 10^{3}$	$3.671\times 10^{3}$	$3.175\times 10^{3}$
	Std	$3.338\times 10^{1}$	$6.318\times 10^{1}$	$8.040\times 10^{1}$	$9.770\times 10^{1}$	$1.457\times 10^{2}$	$6.410\times 10^{1}$	$7.197\times 10^{1}$	$1.339\times 10^{2}$	$1.873\times 10^{2}$	$1.476\times 10^{1}$
F30	Mean	$7.874\times 10^{4}$	$7.173\times 10^{6}$	$8.471\times 10^{6}$	$1.477\times 10^{6}$	$2.127\times 10^{7}$	$6.953\times 10^{5}$	$4.420\times 10^{6}$	$1.017\times 10^{7}$	$3.480\times 10^{7}$	$2.049\times 10^{5}$
	Std	$1.233\times 10^{5}$	$9.826\times 10^{6}$	$8.954\times 10^{6}$	$1.608\times 10^{6}$	$1.885\times 10^{7}$	$9.735\times 10^{5}$	$6.675\times 10^{6}$	$1.103\times 10^{7}$	$2.948\times 10^{7}$	$2.855\times 10^{5}$
Friedman Rank		**1.207**	6.793	8.000	4.897	6.862	2.897	5.655	6.724	9.966	2.000
Final Rank		**1**	7	9	4	8	3	5	6	10	2

**Table 4:** Wilcoxon rank sum test results for CEC2017 in dimension 10.

Fun	ICOA	COA	WOA	AOA	BKA	HOA	NCHHO	ISCSO	LSHADE_cnEpSin
F1	$3.02\times 10^{-11}$	$3.02\times 10^{-11}$	$1.33\times 10^{-10}$	$3.02\times 10^{-11}$	$4.42\times 10^{-06}$	$3.02\times 10^{-11}$	$3.02\times 10^{-11}$	$3.02\times 10^{-11}$	$7.38\times 10^{-10}$
F3	$3.02\times 10^{-11}$	$3.02\times 10^{-11}$	$3.02\times 10^{-11}$	$3.02\times 10^{-11}$	$2.68\times 10^{-06}$	$3.02\times 10^{-11}$	$3.02\times 10^{-11}$	$3.02\times 10^{-11}$	$3.02\times 10^{-11}$
F4	$3.02\times 10^{-11}$	$3.02\times 10^{-11}$	$1.61\times 10^{-10}$	$3.02\times 10^{-11}$	$3.87\times 10^{-01}$	$3.02\times 10^{-11}$	$3.02\times 10^{-11}$	$3.02\times 10^{-11}$	$3.69\times 10^{-11}$
F5	$3.01\times 10^{-11}$	$3.01\times 10^{-11}$	$3.01\times 10^{-11}$	$3.01\times 10^{-11}$	$4.60\times 10^{-10}$	$3.32\times 10^{-11}$	$3.01\times 10^{-11}$	$3.01\times 10^{-11}$	$1.46\times 10^{-10}$
F6	$3.02\times 10^{-11}$	$3.02\times 10^{-11}$	$3.02\times 10^{-11}$	$3.02\times 10^{-11}$	$3.02\times 10^{-11}$	$3.02\times 10^{-11}$	$3.02\times 10^{-11}$	$3.02\times 10^{-11}$	$1.49\times 10^{-06}$
F7	$5.49\times 10^{-11}$	$4.98\times 10^{-11}$	$2.15\times 10^{-10}$	$6.70\times 10^{-11}$	$4.74\times 10^{-06}$	$5.46\times 10^{-09}$	$4.08\times 10^{-11}$	$3.02\times 10^{-11}$	$7.20\times 10^{-05}$
F8	$3.01\times 10^{-11}$	$3.01\times 10^{-11}$	$3.68\times 10^{-11}$	$4.96\times 10^{-11}$	$2.28\times 10^{-05}$	$4.07\times 10^{-11}$	$3.01\times 10^{-11}$	$3.01\times 10^{-11}$	$3.08\times 10^{-08}$
F9	$3.33\times 10^{-11}$	$3.02\times 10^{-11}$	$1.09\times 10^{-10}$	$3.02\times 10^{-11}$	$2.37\times 10^{-10}$	$2.61\times 10^{-10}$	$3.02\times 10^{-11}$	$3.02\times 10^{-11}$	$7.39\times 10^{-01}$
F10	$3.02\times 10^{-11}$	$3.02\times 10^{-11}$	$5.46\times 10^{-09}$	$1.21\times 10^{-10}$	$3.57\times 10^{-06}$	$1.33\times 10^{-10}$	$7.39\times 10^{-11}$	$3.02\times 10^{-11}$	$1.29\times 10^{-09}$
F11	$3.02\times 10^{-11}$	$3.02\times 10^{-11}$	$1.96\times 10^{-10}$	$3.69\times 10^{-11}$	$8.20\times 10^{-07}$	$4.08\times 10^{-11}$	$3.34\times 10^{-11}$	$3.02\times 10^{-11}$	$2.84\times 10^{-04}$
F12	$3.02\times 10^{-11}$	$3.02\times 10^{-11}$	$5.49\times 10^{-11}$	$8.10\times 10^{-10}$	$9.93\times 10^{-02}$	$3.34\times 10^{-11}$	$3.34\times 10^{-11}$	$3.02\times 10^{-11}$	$5.49\times 10^{-11}$
F13	$3.02\times 10^{-11}$	$3.02\times 10^{-11}$	$3.02\times 10^{-11}$	$3.02\times 10^{-11}$	$8.15\times 10^{-11}$	$3.02\times 10^{-11}$	$3.02\times 10^{-11}$	$3.02\times 10^{-11}$	$3.35\times 10^{-08}$
F14	$3.02\times 10^{-11}$	$3.02\times 10^{-11}$	$3.02\times 10^{-11}$	$3.02\times 10^{-11}$	$3.02\times 10^{-11}$	$3.02\times 10^{-11}$	$3.02\times 10^{-11}$	$3.02\times 10^{-11}$	$4.07\times 10^{-11}$
F15	$3.02\times 10^{-11}$	$3.02\times 10^{-11}$	$3.02\times 10^{-11}$	$3.02\times 10^{-11}$	$4.50\times 10^{-11}$	$3.02\times 10^{-11}$	$3.02\times 10^{-11}$	$3.02\times 10^{-11}$	$2.20\times 10^{-07}$
F16	$2.15\times 10^{-10}$	$7.38\times 10^{-10}$	$1.25\times 10^{-04}$	$3.50\times 10^{-09}$	$2.06\times 10^{-01}$	$1.39\times 10^{-06}$	$1.87\times 10^{-07}$	$1.96\times 10^{-10}$	$6.77\times 10^{-05}$
F17	$3.34\times 10^{-11}$	$3.02\times 10^{-11}$	$3.69\times 10^{-11}$	$3.02\times 10^{-11}$	$1.61\times 10^{-10}$	$3.34\times 10^{-11}$	$3.02\times 10^{-11}$	$3.02\times 10^{-11}$	$8.48\times 10^{-09}$
F18	$3.02\times 10^{-11}$	$3.02\times 10^{-11}$	$3.02\times 10^{-11}$	$3.02\times 10^{-11}$	$6.07\times 10^{-11}$	$3.02\times 10^{-11}$	$3.34\times 10^{-11}$	$3.02\times 10^{-11}$	$6.70\times 10^{-11}$
F19	$3.02\times 10^{-11}$	$3.02\times 10^{-11}$	$3.02\times 10^{-11}$	$3.02\times 10^{-11}$	$3.34\times 10^{-11}$	$3.02\times 10^{-11}$	$3.02\times 10^{-11}$	$3.02\times 10^{-11}$	$4.62\times 10^{-10}$
F20	$2.95\times 10^{-11}$	$2.95\times 10^{-11}$	$2.95\times 10^{-11}$	$3.61\times 10^{-11}$	$9.70\times 10^{-11}$	$3.98\times 10^{-11}$	$2.95\times 10^{-11}$	$2.95\times 10^{-11}$	$6.99\times 10^{-09}$
F21	$8.84\times 10^{-07}$	$8.48\times 10^{-09}$	$8.20\times 10^{-07}$	$5.19\times 10^{-07}$	$1.99\times 10^{-02}$	$1.01\times 10^{-08}$	$1.73\times 10^{-07}$	$9.76\times 10^{-10}$	$1.24\times 10^{-03}$
F22	$3.02\times 10^{-11}$	$3.02\times 10^{-11}$	$4.20\times 10^{-10}$	$3.02\times 10^{-11}$	$6.74\times 10^{-06}$	$3.02\times 10^{-11}$	$3.47\times 10^{-10}$	$3.02\times 10^{-11}$	$4.08\times 10^{-11}$
F23	$3.02\times 10^{-11}$	$3.02\times 10^{-11}$	$2.83\times 10^{-08}$	$3.02\times 10^{-11}$	$5.37\times 10^{-02}$	$3.02\times 10^{-11}$	$3.02\times 10^{-11}$	$3.02\times 10^{-11}$	$3.11\times 10^{-01}$
F24	$3.92\times 10^{-11}$	$3.20\times 10^{-11}$	$3.20\times 10^{-11}$	$5.29\times 10^{-11}$	$7.87\times 10^{-11}$	$5.29\times 10^{-11}$	$3.20\times 10^{-11}$	$2.36\times 10^{-11}$	$1.42\times 10^{-10}$
F25	$3.02\times 10^{-11}$	$3.02\times 10^{-11}$	$2.03\times 10^{-07}$	$3.02\times 10^{-11}$	$3.33\times 10^{-01}$	$3.02\times 10^{-11}$	$3.02\times 10^{-11}$	$3.02\times 10^{-11}$	$1.44\times 10^{-02}$
F26	$2.76\times 10^{-11}$	$2.76\times 10^{-11}$	$9.03\times 10^{-10}$	$2.76\times 10^{-11}$	$8.54\times 10^{-08}$	$2.76\times 10^{-11}$	$3.73\times 10^{-11}$	$2.76\times 10^{-11}$	$9.06\times 10^{-07}$
F27	$3.02\times 10^{-11}$	$3.02\times 10^{-11}$	$3.47\times 10^{-10}$	$3.02\times 10^{-11}$	$2.53\times 10^{-04}$	$3.02\times 10^{-11}$	$3.02\times 10^{-11}$	$3.02\times 10^{-11}$	$7.74\times 10^{-06}$
F28	$3.02\times 10^{-11}$	$3.02\times 10^{-11}$	$3.56\times 10^{-04}$	$1.61\times 10^{-10}$	$8.30\times 10^{-01}$	$1.78\times 10^{-10}$	$1.19\times 10^{-06}$	$3.02\times 10^{-11}$	$2.12\times 10^{-01}$
F29	$3.02\times 10^{-11}$	$3.02\times 10^{-11}$	$2.23\times 10^{-09}$	$2.61\times 10^{-10}$	$1.39\times 10^{-06}$	$3.82\times 10^{-10}$	$1.86\times 10^{-09}$	$3.69\times 10^{-11}$	$2.77\times 10^{-01}$
F30	$3.33\times 10^{-11}$	$3.02\times 10^{-11}$	$1.10\times 10^{-08}$	$3.02\times 10^{-11}$	$9.47\times 10^{-01}$	$7.39\times 10^{-11}$	$4.50\times 10^{-11}$	$3.34\times 10^{-11}$	$2.61\times 10^{-02}$
+/=/−	29/0/0	29/0/0	29/0/0	29/0/0	22/7/0	29/0/0	29/0/0	29/0/0	23/4/2

**Table 5:** Results of CEC2017 in dimension 30.

Fun	Index	MSECOA	ICOA	COA	WOA	AOA	BKA	HOA	NCHHO	ISCSO	LSHADE_cnEpSin
F1	Mean	$1.028\times 10^{8}$	$5.326\times 10^{10}$	$5.664\times 10^{10}$	$1.406\times 10^{9}$	$5.339\times 10^{10}$	$1.129\times 10^{10}$	$3.534\times 10^{10}$	$4.159\times 10^{10}$	$6.315\times 10^{10}$	$1.221\times 10^{9}$
	Std	$3.275\times 10^{8}$	$5.763\times 10^{9}$	$7.837\times 10^{9}$	$6.061\times 10^{8}$	$1.112\times 10^{10}$	$1.655\times 10^{10}$	$7.570\times 10^{9}$	$5.719\times 10^{9}$	$7.372\times 10^{9}$	$3.678\times 10^{8}$
F3	Mean	$5.322\times 10^{4}$	$8.419\times 10^{4}$	$8.376\times 10^{4}$	$2.598\times 10^{5}$	$8.272\times 10^{4}$	$2.647\times 10^{4}$	$6.992\times 10^{4}$	$8.506\times 10^{4}$	$6.564\times 10^{5}$	$1.106\times 10^{5}$
	Std	$8.793\times 10^{3}$	$4.570\times 10^{3}$	$5.428\times 10^{3}$	$8.634\times 10^{4}$	$9.602\times 10^{3}$	$1.903\times 10^{4}$	$7.800\times 10^{3}$	$4.538\times 10^{3}$	$2.999\times 10^{6}$	$2.161\times 10^{4}$
F4	Mean	$5.305\times 10^{2}$	$1.525\times 10^{4}$	$1.579\times 10^{4}$	$8.734\times 10^{2}$	$1.396\times 10^{4}$	$1.561\times 10^{3}$	$7.553\times 10^{3}$	$1.049\times 10^{4}$	$1.896\times 10^{4}$	$5.703\times 10^{2}$
	Std	$3.465\times 10^{1}$	$2.210\times 10^{3}$	$2.824\times 10^{3}$	$1.268\times 10^{2}$	$4.590\times 10^{3}$	$3.152\times 10^{3}$	$1.656\times 10^{3}$	$2.086\times 10^{3}$	$3.009\times 10^{3}$	$2.952\times 10^{1}$
F5	Mean	$7.024\times 10^{2}$	$9.326\times 10^{2}$	$9.197\times 10^{2}$	$8.311\times 10^{2}$	$8.718\times 10^{2}$	$7.371\times 10^{2}$	$8.136\times 10^{2}$	$8.993\times 10^{2}$	$9.757\times 10^{2}$	$7.271\times 10^{2}$
	Std	$4.472\times 10^{1}$	$2.192\times 10^{1}$	$2.562\times 10^{1}$	$5.002\times 10^{1}$	$4.565\times 10^{1}$	$4.871\times 10^{1}$	$3.903\times 10^{1}$	$2.982\times 10^{1}$	$3.262\times 10^{1}$	$1.346\times 10^{1}$
F6	Mean	$6.166\times 10^{2}$	$6.882\times 10^{2}$	$6.903\times 10^{2}$	$6.785\times 10^{2}$	$6.771\times 10^{2}$	$6.606\times 10^{2}$	$6.661\times 10^{2}$	$6.847\times 10^{2}$	$7.049\times 10^{2}$	$6.209\times 10^{2}$
	Std	$7.433\times 10^{0}$	$7.063\times 10^{0}$	$6.806\times 10^{0}$	$1.243\times 10^{1}$	$8.986\times 10^{0}$	$9.221\times 10^{0}$	$8.131\times 10^{0}$	$9.429\times 10^{0}$	$9.173\times 10^{0}$	$4.280\times 10^{0}$
F7	Mean	$9.985\times 10^{2}$	$1.385\times 10^{3}$	$1.429\times 10^{3}$	$1.281\times 10^{3}$	$1.364\times 10^{3}$	$1.228\times 10^{3}$	$1.244\times 10^{3}$	$1.389\times 10^{3}$	$1.499\times 10^{3}$	$1.028\times 10^{3}$
	Std	$6.922\times 10^{1}$	$3.950\times 10^{1}$	$4.740\times 10^{1}$	$7.172\times 10^{1}$	$5.629\times 10^{1}$	$6.611\times 10^{1}$	$7.358\times 10^{1}$	$3.248\times 10^{1}$	$6.541\times 10^{1}$	$2.130\times 10^{1}$
F8	Mean	$9.507\times 10^{2}$	$1.144\times 10^{3}$	$1.146\times 10^{3}$	$1.048\times 10^{3}$	$1.119\times 10^{3}$	$9.852\times 10^{2}$	$1.068\times 10^{3}$	$1.111\times 10^{3}$	$1.195\times 10^{3}$	$1.022\times 10^{3}$
	Std	$2.283\times 10^{1}$	$1.595\times 10^{1}$	$2.909\times 10^{1}$	$4.816\times 10^{1}$	$3.481\times 10^{1}$	$5.531\times 10^{1}$	$3.254\times 10^{1}$	$2.540\times 10^{1}$	$3.551\times 10^{1}$	$1.651\times 10^{1}$
F9	Mean	$3.073\times 10^{3}$	$1.103\times 10^{4}$	$1.102\times 10^{4}$	$1.090\times 10^{4}$	$7.250\times 10^{3}$	$5.447\times 10^{3}$	$7.080\times 10^{3}$	$9.209\times 10^{3}$	$1.518\times 10^{4}$	$3.429\times 10^{3}$
	Std	$1.132\times 10^{3}$	$1.277\times 10^{3}$	$1.721\times 10^{3}$	$4.350\times 10^{3}$	$1.121\times 10^{3}$	$1.339\times 10^{3}$	$1.172\times 10^{3}$	$1.099\times 10^{3}$	$2.141\times 10^{3}$	$6.441\times 10^{2}$
F10	Mean	$4.935\times 10^{3}$	$8.960\times 10^{3}$	$8.955\times 10^{3}$	$7.225\times 10^{3}$	$7.433\times 10^{3}$	$5.137\times 10^{3}$	$7.480\times 10^{3}$	$8.466\times 10^{3}$	$9.786\times 10^{3}$	$8.152\times 10^{3}$
	Std	$7.199\times 10^{2}$	$3.565\times 10^{2}$	$3.334\times 10^{2}$	$8.959\times 10^{2}$	$6.233\times 10^{2}$	$7.096\times 10^{2}$	$5.834\times 10^{2}$	$5.703\times 10^{2}$	$7.368\times 10^{2}$	$3.755\times 10^{2}$
F11	Mean	$1.272\times 10^{3}$	$8.813\times 10^{3}$	$8.378\times 10^{3}$	$7.656\times 10^{3}$	$9.637\times 10^{3}$	$1.722\times 10^{3}$	$6.525\times 10^{3}$	$9.266\times 10^{3}$	$2.068\times 10^{4}$	$1.860\times 10^{3}$
	Std	$6.087\times 10^{1}$	$1.709\times 10^{3}$	$2.021\times 10^{3}$	$3.316\times 10^{3}$	$3.200\times 10^{3}$	$1.189\times 10^{3}$	$1.967\times 10^{3}$	$1.803\times 10^{3}$	$1.305\times 10^{4}$	$1.416\times 10^{2}$
F12	Mean	$1.661\times 10^{6}$	$1.165\times 10^{10}$	$1.260\times 10^{10}$	$2.998\times 10^{8}$	$1.430\times 10^{10}$	$5.199\times 10^{8}$	$5.896\times 10^{9}$	$8.991\times 10^{9}$	$1.886\times 10^{10}$	$8.299\times 10^{7}$
	Std	$1.205\times 10^{6}$	$2.786\times 10^{9}$	$3.398\times 10^{9}$	$2.415\times 10^{8}$	$3.355\times 10^{9}$	$1.805\times 10^{9}$	$1.660\times 10^{9}$	$3.376\times 10^{9}$	$4.806\times 10^{9}$	$2.379\times 10^{7}$
F13	Mean	$9.409\times 10^{3}$	$6.922\times 10^{9}$	$9.887\times 10^{9}$	$2.512\times 10^{6}$	$1.174\times 10^{10}$	$1.756\times 10^{5}$	$2.913\times 10^{9}$	$3.518\times 10^{9}$	$1.788\times 10^{10}$	$1.188\times 10^{7}$
	Std	$5.455\times 10^{3}$	$4.074\times 10^{9}$	$5.511\times 10^{9}$	$3.546\times 10^{6}$	$4.683\times 10^{9}$	$1.039\times 10^{5}$	$1.869\times 10^{9}$	$4.045\times 10^{9}$	$7.519\times 10^{9}$	$6.959\times 10^{6}$
F14	Mean	$2.348\times 10^{4}$	$4.742\times 10^{6}$	$3.987\times 10^{6}$	$2.171\times 10^{6}$	$2.358\times 10^{6}$	$5.706\times 10^{4}$	$2.115\times 10^{6}$	$3.603\times 10^{6}$	$4.424\times 10^{7}$	$9.505\times 10^{4}$
	Std	$6.089\times 10^{4}$	$3.690\times 10^{6}$	$4.156\times 10^{6}$	$2.705\times 10^{6}$	$2.234\times 10^{6}$	$1.856\times 10^{5}$	$2.241\times 10^{6}$	$2.943\times 10^{6}$	$4.890\times 10^{7}$	$5.265\times 10^{4}$
F15	Mean	$3.694\times 10^{3}$	$6.582\times 10^{8}$	$7.717\times 10^{8}$	$1.170\times 10^{6}$	$2.945\times 10^{6}$	$1.344\times 10^{5}$	$9.781\times 10^{7}$	$2.291\times 10^{8}$	$3.199\times 10^{9}$	$9.038\times 10^{5}$
	Std	$3.223\times 10^{3}$	$3.295\times 10^{8}$	$5.643\times 10^{8}$	$1.585\times 10^{6}$	$7.510\times 10^{6}$	$4.670\times 10^{5}$	$9.557\times 10^{7}$	$2.613\times 10^{8}$	$1.165\times 10^{9}$	$5.529\times 10^{5}$
F16	Mean	$2.558\times 10^{3}$	$5.514\times 10^{3}$	$6.240\times 10^{3}$	$4.324\times 10^{3}$	$5.229\times 10^{3}$	$3.136\times 10^{3}$	$4.625\times 10^{3}$	$4.969\times 10^{3}$	$7.573\times 10^{3}$	$3.173\times 10^{3}$
	Std	$3.115\times 10^{2}$	$1.024\times 10^{3}$	$1.148\times 10^{3}$	$6.969\times 10^{2}$	$1.305\times 10^{3}$	$5.254\times 10^{2}$	$7.132\times 10^{2}$	$8.859\times 10^{2}$	$2.587\times 10^{3}$	$1.943\times 10^{2}$
F17	Mean	$2.107\times 10^{3}$	$3.461\times 10^{3}$	$4.465\times 10^{3}$	$2.654\times 10^{3}$	$4.110\times 10^{3}$	$2.347\times 10^{3}$	$3.001\times 10^{3}$	$3.694\times 10^{3}$	$1.710\times 10^{4}$	$2.260\times 10^{3}$
	Std	$1.991\times 10^{2}$	$4.300\times 10^{2}$	$2.139\times 10^{3}$	$3.061\times 10^{2}$	$1.187\times 10^{3}$	$2.953\times 10^{2}$	$4.827\times 10^{2}$	$2.163\times 10^{3}$	$2.034\times 10^{4}$	$1.329\times 10^{2}$
F18	Mean	$3.085\times 10^{5}$	$4.029\times 10^{7}$	$5.965\times 10^{7}$	$7.200\times 10^{6}$	$2.259\times 10^{7}$	$1.644\times 10^{5}$	$1.650\times 10^{7}$	$5.686\times 10^{7}$	$3.522\times 10^{8}$	$3.256\times 10^{6}$
	Std	$4.078\times 10^{5}$	$3.564\times 10^{7}$	$5.202\times 10^{7}$	$7.166\times 10^{6}$	$1.549\times 10^{7}$	$2.173\times 10^{5}$	$1.648\times 10^{7}$	$6.628\times 10^{7}$	$3.358\times 10^{8}$	$1.669\times 10^{6}$
F19	Mean	$7.628\times 10^{3}$	$7.142\times 10^{8}$	$5.809\times 10^{8}$	$1.015\times 10^{7}$	$1.722\times 10^{7}$	$1.067\times 10^{6}$	$5.514\times 10^{7}$	$3.404\times 10^{8}$	$2.741\times 10^{9}$	$1.301\times 10^{6}$
	Std	$9.997\times 10^{3}$	$3.955\times 10^{8}$	$4.234\times 10^{8}$	$1.020\times 10^{7}$	$6.744\times 10^{7}$	$3.719\times 10^{6}$	$8.796\times 10^{7}$	$2.742\times 10^{8}$	$9.831\times 10^{8}$	$7.965\times 10^{5}$
F20	Mean	$2.358\times 10^{3}$	$3.098\times 10^{3}$	$3.052\times 10^{3}$	$2.867\times 10^{3}$	$2.784\times 10^{3}$	$2.516\times 10^{3}$	$2.765\times 10^{3}$	$2.979\times 10^{3}$	$3.495\times 10^{3}$	$2.510\times 10^{3}$
	Std	$1.261\times 10^{2}$	$1.590\times 10^{2}$	$2.301\times 10^{2}$	$1.962\times 10^{2}$	$1.585\times 10^{2}$	$1.620\times 10^{2}$	$1.707\times 10^{2}$	$2.066\times 10^{2}$	$2.274\times 10^{2}$	$1.162\times 10^{2}$
F21	Mean	$2.441\times 10^{3}$	$2.743\times 10^{3}$	$2.745\times 10^{3}$	$2.632\times 10^{3}$	$2.679\times 10^{3}$	$2.543\times 10^{3}$	$2.608\times 10^{3}$	$2.743\times 10^{3}$	$2.836\times 10^{3}$	$2.517\times 10^{3}$
	Std	$3.085\times 10^{1}$	$4.065\times 10^{1}$	$5.397\times 10^{1}$	$7.172\times 10^{1}$	$5.629\times 10^{1}$	$6.622\times 10^{1}$	$3.813\times 10^{1}$	$5.550\times 10^{1}$	$7.803\times 10^{1}$	$1.507\times 10^{1}$
F22	Mean	$2.452\times 10^{3}$	$9.442\times 10^{3}$	$9.739\times 10^{3}$	$8.224\times 10^{3}$	$9.065\times 10^{3}$	$6.187\times 10^{3}$	$7.609\times 10^{3}$	$9.513\times 10^{3}$	$1.071\times 10^{4}$	$3.597\times 10^{3}$
	Std	$7.149\times 10^{2}$	$6.350\times 10^{2}$	$6.191\times 10^{2}$	$7.062\times 10^{2}$	$6.224\times 10^{2}$	$1.770\times 10^{3}$	$1.309\times 10^{3}$	$6.843\times 10^{2}$	$7.697\times 10^{2}$	$3.047\times 10^{2}$
F23	Mean	$2.785\times 10^{3}$	$3.446\times 10^{3}$	$3.631\times 10^{3}$	$3.130\times 10^{3}$	$3.443\times 10^{3}$	$3.124\times 10^{3}$	$3.444\times 10^{3}$	$3.414\times 10^{3}$	$3.737\times 10^{3}$	$2.865\times 10^{3}$
	Std	$3.909\times 10^{1}$	$1.536\times 10^{2}$	$1.607\times 10^{2}$	$1.035\times 10^{2}$	$1.426\times 10^{2}$	$9.757\times 10^{1}$	$1.086\times 10^{2}$	$1.650\times 10^{2}$	$2.693\times 10^{2}$	$1.598\times 10^{1}$
F24	Mean	$3.007\times 10^{3}$	$3.668\times 10^{3}$	$3.821\times 10^{3}$	$3.231\times 10^{3}$	$3.859\times 10^{3}$	$3.296\times 10^{3}$	$3.806\times 10^{3}$	$3.684\times 10^{3}$	$4.037\times 10^{3}$	$3.023\times 10^{3}$
	Std	$5.952\times 10^{1}$	$1.356\times 10^{2}$	$1.768\times 10^{2}$	$9.318\times 10^{1}$	$2.103\times 10^{2}$	$1.295\times 10^{2}$	$1.412\times 10^{2}$	$2.137\times 10^{2}$	$3.031\times 10^{2}$	$2.099\times 10^{1}$
F25	Mean	$2.936\times 10^{3}$	$4.919\times 10^{3}$	$5.115\times 10^{3}$	$3.115\times 10^{3}$	$5.656\times 10^{3}$	$3.288\times 10^{3}$	$3.845\times 10^{3}$	$4.388\times 10^{3}$	$6.321\times 10^{3}$	$2.980\times 10^{3}$
	Std	$2.883\times 10^{1}$	$3.494\times 10^{2}$	$4.132\times 10^{2}$	$5.708\times 10^{1}$	$8.271\times 10^{2}$	$6.449\times 10^{2}$	$2.339\times 10^{2}$	$3.940\times 10^{2}$	$4.048\times 10^{2}$	$1.936\times 10^{1}$
F26	Mean	$4.767\times 10^{3}$	$1.110\times 10^{4}$	$1.196\times 10^{4}$	$8.384\times 10^{3}$	$1.037\times 10^{4}$	$7.517\times 10^{3}$	$9.460\times 10^{3}$	$1.082\times 10^{4}$	$1.283\times 10^{4}$	$5.846\times 10^{3}$
	Std	$1.621\times 10^{3}$	$7.347\times 10^{2}$	$9.633\times 10^{2}$	$9.450\times 10^{2}$	$9.525\times 10^{2}$	$1.740\times 10^{3}$	$7.934\times 10^{2}$	$8.885\times 10^{2}$	$1.258\times 10^{3}$	$1.775\times 10^{2}$
F27	Mean	$3.307\times 10^{3}$	$4.345\times 10^{3}$	$4.689\times 10^{3}$	$3.473\times 10^{3}$	$4.543\times 10^{3}$	$3.408\times 10^{3}$	$4.340\times 10^{3}$	$4.237\times 10^{3}$	$4.873\times 10^{3}$	$3.227\times 10^{3}$
	Std	$3.850\times 10^{1}$	$3.063\times 10^{2}$	$3.778\times 10^{2}$	$1.309\times 10^{2}$	$2.853\times 10^{2}$	$1.205\times 10^{2}$	$3.365\times 10^{2}$	$4.410\times 10^{2}$	$6.351\times 10^{2}$	$4.235\times 10^{0}$
F28	Mean	$3.305\times 10^{3}$	$6.938\times 10^{3}$	$7.650\times 10^{3}$	$3.557\times 10^{3}$	$7.131\times 10^{3}$	$3.551\times 10^{3}$	$5.866\times 10^{3}$	$6.370\times 10^{3}$	$8.680\times 10^{3}$	$3.426\times 10^{3}$
	Std	$3.556\times 10^{1}$	$6.543\times 10^{2}$	$7.200\times 10^{2}$	$9.222\times 10^{1}$	$9.329\times 10^{2}$	$5.798\times 10^{2}$	$5.881\times 10^{2}$	$5.108\times 10^{2}$	$4.614\times 10^{2}$	$5.654\times 10^{1}$
F29	Mean	$3.906\times 10^{3}$	$7.719\times 10^{3}$	$8.635\times 10^{3}$	$5.308\times 10^{3}$	$7.236\times 10^{3}$	$4.668\times 10^{3}$	$6.254\times 10^{3}$	$6.799\times 10^{3}$	$1.337\times 10^{4}$	$4.246\times 10^{3}$
	Std	$2.728\times 10^{2}$	$1.103\times 10^{3}$	$1.851\times 10^{3}$	$5.253\times 10^{2}$	$1.998\times 10^{3}$	$3.951\times 10^{2}$	$1.035\times 10^{3}$	$1.124\times 10^{3}$	$7.988\times 10^{3}$	$1.953\times 10^{2}$
F30	Mean	$4.702\times 10^{4}$	$1.169\times 10^{9}$	$1.790\times 10^{9}$	$3.906\times 10^{7}$	$1.668\times 10^{9}$	$3.100\times 10^{6}$	$6.171\times 10^{8}$	$5.951\times 10^{8}$	$2.082\times 10^{9}$	$9.276\times 10^{5}$
	Std	$5.467\times 10^{4}$	$5.227\times 10^{8}$	$1.002\times 10^{9}$	$2.719\times 10^{7}$	$9.835\times 10^{8}$	$8.717\times 10^{6}$	$4.662\times 10^{8}$	$3.589\times 10^{8}$	$2.015\times 10^{9}$	$5.297\times 10^{5}$
Friedman Rank		**1.103**	7.552	8.414	4.379	6.690	2.759	4.897	6.586	10.000	2.621
Final Rank		**1**	8	9	4	7	3	5	6	10	2

**Table 6:** Wilcoxon rank sum test results for CEC2017 in dimension 30.

Fun	ICOA	COA	WOA	AOA	BKA	HOA	NCHHO	ISCSO	LSHADE_cnEpSin
F1	$3.02\times 10^{-11}$	$3.02\times 10^{-11}$	$4.20\times 10^{-10}$	$3.02\times 10^{-11}$	$2.61\times 10^{-10}$	$3.02\times 10^{-11}$	$3.02\times 10^{-11}$	$3.02\times 10^{-11}$	$5.57\times 10^{-10}$
F3	$3.02\times 10^{-11}$	$3.02\times 10^{-11}$	$3.02\times 10^{-11}$	$1.46\times 10^{-10}$	$8.84\times 10^{-7}$	$1.70\times 10^{-8}$	$3.02\times 10^{-11}$	$3.02\times 10^{-11}$	$3.34\times 10^{-11}$
F4	$3.02\times 10^{-11}$	$3.02\times 10^{-11}$	$3.02\times 10^{-11}$	$3.02\times 10^{-11}$	$1.61\times 10^{-6}$	$3.02\times 10^{-11}$	$3.02\times 10^{-11}$	$3.02\times 10^{-11}$	$6.77\times 10^{-5}$
F5	$3.02\times 10^{-11}$	$3.02\times 10^{-11}$	$1.21\times 10^{-10}$	$3.02\times 10^{-11}$	$6.38\times 10^{-3}$	$2.87\times 10^{-10}$	$3.02\times 10^{-11}$	$3.02\times 10^{-11}$	$1.76\times 10^{-2}$
F6	$3.02\times 10^{-11}$	$3.02\times 10^{-11}$	$3.02\times 10^{-11}$	$3.02\times 10^{-11}$	$3.02\times 10^{-11}$	$3.02\times 10^{-11}$	$3.02\times 10^{-11}$	$3.02\times 10^{-11}$	$1.33\times 10^{-2}$
F7	$3.02\times 10^{-11}$	$3.02\times 10^{-11}$	$3.02\times 10^{-11}$	$3.02\times 10^{-11}$	$3.69\times 10^{-11}$	$4.08\times 10^{-11}$	$3.02\times 10^{-11}$	$3.02\times 10^{-11}$	$4.51\times 10^{-2}$
F8	$3.02\times 10^{-11}$	$3.02\times 10^{-11}$	$9.92\times 10^{-11}$	$3.02\times 10^{-11}$	$1.17\times 10^{-3}$	$3.02\times 10^{-11}$	$3.02\times 10^{-11}$	$3.02\times 10^{-11}$	$4.98\times 10^{-11}$
F9	$3.02\times 10^{-11}$	$3.02\times 10^{-11}$	$4.50\times 10^{-11}$	$9.92\times 10^{-11}$	$1.43\times 10^{-8}$	$2.15\times 10^{-10}$	$3.02\times 10^{-11}$	$3.02\times 10^{-11}$	$2.81\times 10^{-2}$
F10	$3.02\times 10^{-11}$	$3.02\times 10^{-11}$	$5.49\times 10^{-11}$	$3.02\times 10^{-11}$	$4.38\times 10^{0}$	$3.02\times 10^{-11}$	$3.02\times 10^{-11}$	$3.02\times 10^{-11}$	$3.02\times 10^{-11}$
F11	$3.02\times 10^{-11}$	$3.02\times 10^{-11}$	$3.02\times 10^{-11}$	$3.02\times 10^{-11}$	$3.16\times 10^{-5}$	$3.02\times 10^{-11}$	$3.02\times 10^{-11}$	$3.02\times 10^{-11}$	$3.02\times 10^{-11}$
F12	$3.02\times 10^{-11}$	$3.02\times 10^{-11}$	$3.02\times 10^{-11}$	$3.02\times 10^{-11}$	$2.03\times 10^{-9}$	$3.02\times 10^{-11}$	$3.02\times 10^{-11}$	$3.02\times 10^{-11}$	$3.02\times 10^{-11}$
F13	$3.02\times 10^{-11}$	$3.02\times 10^{-11}$	$3.02\times 10^{-11}$	$3.02\times 10^{-11}$	$3.02\times 10^{-11}$	$3.02\times 10^{-11}$	$3.02\times 10^{-11}$	$3.02\times 10^{-11}$	$3.02\times 10^{-11}$
F14	$3.69\times 10^{-11}$	$3.69\times 10^{-11}$	$4.62\times 10^{-10}$	$8.15\times 10^{-11}$	$7.62\times 10^{0}$	$3.34\times 10^{-11}$	$3.02\times 10^{-11}$	$3.02\times 10^{-11}$	$1.20\times 10^{-8}$
F15	$3.02\times 10^{-11}$	$3.02\times 10^{-11}$	$3.02\times 10^{-11}$	$4.08\times 10^{-11}$	$3.69\times 10^{-11}$	$3.02\times 10^{-11}$	$3.02\times 10^{-11}$	$3.02\times 10^{-11}$	$3.02\times 10^{-11}$
F16	$3.02\times 10^{-11}$	$3.02\times 10^{-11}$	$3.02\times 10^{-11}$	$3.02\times 10^{-11}$	$6.53\times 10^{-7}$	$3.02\times 10^{-11}$	$3.02\times 10^{-11}$	$3.02\times 10^{-11}$	$2.23\times 10^{-9}$
F17	$3.02\times 10^{-11}$	$3.02\times 10^{-11}$	$2.60\times 10^{-8}$	$4.50\times 10^{-11}$	$1.17\times 10^{-3}$	$1.61\times 10^{-10}$	$8.15\times 10^{-11}$	$3.02\times 10^{-11}$	$5.87\times 10^{-4}$
F18	$3.02\times 10^{-11}$	$4.98\times 10^{-11}$	$3.47\times 10^{-10}$	$3.02\times 10^{-11}$	$2.61\times 10^{0}$	$4.08\times 10^{-11}$	$4.08\times 10^{-11}$	$3.02\times 10^{-11}$	$5.49\times 10^{-11}$
F19	$3.02\times 10^{-11}$	$3.02\times 10^{-11}$	$3.02\times 10^{-11}$	$3.02\times 10^{-11}$	$6.12\times 10^{-10}$	$3.02\times 10^{-11}$	$3.02\times 10^{-11}$	$3.02\times 10^{-11}$	$3.02\times 10^{-11}$
F20	$3.02\times 10^{-11}$	$1.09\times 10^{-10}$	$6.07\times 10^{-11}$	$4.62\times 10^{-10}$	$5.26\times 10^{-4}$	$1.96\times 10^{-10}$	$4.08\times 10^{-11}$	$3.02\times 10^{-11}$	$2.00\times 10^{-5}$
F21	$3.02\times 10^{-11}$	$3.02\times 10^{-11}$	$3.69\times 10^{-11}$	$3.02\times 10^{-11}$	$1.86\times 10^{-9}$	$3.02\times 10^{-11}$	$3.02\times 10^{-11}$	$3.02\times 10^{-11}$	$4.98\times 10^{-11}$
F22	$3.02\times 10^{-11}$	$3.02\times 10^{-11}$	$3.02\times 10^{-11}$	$3.02\times 10^{-11}$	$8.99\times 10^{-11}$	$4.50\times 10^{-11}$	$3.02\times 10^{-11}$	$3.02\times 10^{-11}$	$5.57\times 10^{-10}$
F23	$3.02\times 10^{-11}$	$3.02\times 10^{-11}$	$3.02\times 10^{-11}$	$3.02\times 10^{-11}$	$3.02\times 10^{-11}$	$3.02\times 10^{-11}$	$3.02\times 10^{-11}$	$3.02\times 10^{-11}$	$1.61\times 10^{-10}$
F24	$3.02\times 10^{-11}$	$3.02\times 10^{-11}$	$1.61\times 10^{-10}$	$3.02\times 10^{-11}$	$8.15\times 10^{-11}$	$3.02\times 10^{-11}$	$3.02\times 10^{-11}$	$3.02\times 10^{-11}$	$7.98\times 10^{-2}$
F25	$3.02\times 10^{-11}$	$3.02\times 10^{-11}$	$3.02\times 10^{-11}$	$3.02\times 10^{-11}$	$4.18\times 10^{-9}$	$3.02\times 10^{-11}$	$3.02\times 10^{-11}$	$3.02\times 10^{-11}$	$1.36\times 10^{-7}$
F26	$3.02\times 10^{-11}$	$3.02\times 10^{-11}$	$1.09\times 10^{-10}$	$3.02\times 10^{-11}$	$3.52\times 10^{-7}$	$3.02\times 10^{-11}$	$3.02\times 10^{-11}$	$3.02\times 10^{-11}$	$5.75\times 10^{-2}$
F27	$3.02\times 10^{-11}$	$3.02\times 10^{-11}$	$2.03\times 10^{-9}$	$3.02\times 10^{-11}$	$2.84\times 10^{-4}$	$3.02\times 10^{-11}$	$3.02\times 10^{-11}$	$3.02\times 10^{-11}$	$3.34\times 10^{-11}$
F28	$3.02\times 10^{-11}$	$3.02\times 10^{-11}$	$4.50\times 10^{-11}$	$3.02\times 10^{-11}$	$4.74\times 10^{-6}$	$3.02\times 10^{-11}$	$3.02\times 10^{-11}$	$3.02\times 10^{-11}$	$2.37\times 10^{-10}$
F29	$3.02\times 10^{-11}$	$3.02\times 10^{-11}$	$8.15\times 10^{-11}$	$3.02\times 10^{-11}$	$2.23\times 10^{-9}$	$3.02\times 10^{-11}$	$3.02\times 10^{-11}$	$3.02\times 10^{-11}$	$1.34\times 10^{-5}$
F30	$3.02\times 10^{-11}$	$3.02\times 10^{-11}$	$3.02\times 10^{-11}$	$3.02\times 10^{-11}$	$3.34\times 10^{-11}$	$3.02\times 10^{-11}$	$3.02\times 10^{-11}$	$3.02\times 10^{-11}$	$4.08\times 10^{-11}$
+/=/−	29/0/0	29/0/0	29/0/0	29/0/0	25/2/2	29/0/0	29/0/0	29/0/0	26/2/1

**Table 7:** IEEE CEC2022 benchmark functions.

Type	Function No	Function Description	Range	Optimum
Unimodal	F1	Shifted and full Rotated Zakharov Function	[−100, 100]	300
Basic	F2	Shifted and full Rotated Rosenbrock’s Function	[−100, 100]	400
Basic	F3	Shifted and full Rotated Expanded Schaffer’s f6 Function	[−100, 100]	600
Basic	F4	Shifted and full Rotated Non-Continuous Rastrigin’s Function	[−100, 100]	800
Basic	F5	Shifted and full Rotated Levy Function	[−100, 100]	900
Hybrid	F6	Hybrid Function 1 (*N* = 3)	[−100, 100]	1800
Hybrid	F7	Hybrid Function 2 (*N* = 6)	[−100, 100]	2000
Hybrid	F8	Hybrid Function 3 (*N* = 5)	[−100, 100]	2200
Composition	F9	Composition Function 1 (*N* = 5)	[−100, 100]	2300
Composition	F10	Composition Function 2 (*N* = 4)	[−100, 100]	2400
Composition	F11	Composition Function 3 (*N* = 5)	[−100, 100]	2600
Composition	F12	Composition Function 4 (*N* = 6)	[−100, 100]	2700

**Table 8:** Results of CEC2022 in dimension 20.

Fun	Index	MSECOA	ICOA	COA	WOA	AOA	BKA	HOA	NCHHO	ISCSO	LSHADE_cnEpSin
F1	Mean	$1.402\times 10^{4}$	$4.292\times 10^{4}$	$4.774\times 10^{4}$	$2.374\times 10^{4}$	$3.519\times 10^{4}$	$3.491\times 10^{3}$	$2.943\times 10^{4}$	$5.055\times 10^{4}$	$1.341\times 10^{5}$	$2.254\times 10^{4}$
	Std	$4.170\times 10^{3}$	$1.032\times 10^{4}$	$1.525\times 10^{4}$	$7.447\times 10^{3}$	$9.868\times 10^{3}$	$6.049\times 10^{3}$	$8.750\times 10^{3}$	$2.051\times 10^{4}$	$3.703\times 10^{4}$	$4.958\times 10^{3}$
F2	Mean	$4.704\times 10^{2}$	$2.585\times 10^{3}$	$2.967\times 10^{3}$	$5.756\times 10^{2}$	$2.133\times 10^{3}$	$5.908\times 10^{2}$	$1.426\times 10^{3}$	$1.893\times 10^{3}$	$4.089\times 10^{3}$	$4.581\times 10^{2}$
	Std	$2.903\times 10^{1}$	$4.120\times 10^{2}$	$6.782\times 10^{2}$	$5.738\times 10^{1}$	$6.894\times 10^{2}$	$4.511\times 10^{2}$	$3.485\times 10^{2}$	$5.731\times 10^{2}$	$1.071\times 10^{3}$	$3.789\times 10^{0}$
F3	Mean	$6.052\times 10^{2}$	$6.791\times 10^{2}$	$6.796\times 10^{2}$	$6.735\times 10^{2}$	$6.656\times 10^{2}$	$6.509\times 10^{2}$	$6.549\times 10^{2}$	$6.796\times 10^{2}$	$7.000\times 10^{2}$	$6.100\times 10^{2}$
	Std	$4.615\times 10^{0}$	$9.958\times 10^{0}$	$1.118\times 10^{1}$	$1.297\times 10^{1}$	$7.953\times 10^{0}$	$9.870\times 10^{0}$	$1.030\times 10^{1}$	$9.998\times 10^{0}$	$1.135\times 10^{1}$	$1.786\times 10^{0}$
F4	Mean	$8.690\times 10^{2}$	$9.784\times 10^{2}$	$9.760\times 10^{2}$	$9.267\times 10^{2}$	$9.533\times 10^{2}$	$8.821\times 10^{2}$	$9.184\times 10^{2}$	$9.540\times 10^{2}$	$1.005\times 10^{3}$	$9.178\times 10^{2}$
	Std	$1.142\times 10^{1}$	$1.052\times 10^{1}$	$1.745\times 10^{1}$	$3.776\times 10^{1}$	$1.684\times 10^{1}$	$3.032\times 10^{1}$	$1.789\times 10^{1}$	$1.829\times 10^{1}$	$2.039\times 10^{1}$	$1.559\times 10^{1}$
F5	Mean	$1.683\times 10^{3}$	$3.569\times 10^{3}$	$3.452\times 10^{3}$	$4.048\times 10^{3}$	$2.805\times 10^{3}$	$2.111\times 10^{3}$	$2.584\times 10^{3}$	$3.175\times 10^{3}$	$5.206\times 10^{3}$	$1.427\times 10^{3}$
	Std	$4.609\times 10^{2}$	$3.405\times 10^{2}$	$3.554\times 10^{2}$	$1.285\times 10^{3}$	$4.509\times 10^{2}$	$3.837\times 10^{2}$	$4.835\times 10^{2}$	$2.919\times 10^{2}$	$6.203\times 10^{2}$	$1.816\times 10^{2}$
F6	Mean	$1.155\times 10^{4}$	$2.357\times 10^{9}$	$2.543\times 10^{9}$	$1.323\times 10^{6}$	$7.190\times 10^{8}$	$4.579\times 10^{7}$	$5.043\times 10^{8}$	$9.572\times 10^{8}$	$3.792\times 10^{9}$	$8.300\times 10^{6}$
	Std	$3.205\times 10^{4}$	$7.755\times 10^{8}$	$1.262\times 10^{9}$	$2.311\times 10^{6}$	$7.046\times 10^{8}$	$2.498\times 10^{8}$	$5.384\times 10^{8}$	$7.142\times 10^{8}$	$1.881\times 10^{9}$	$5.867\times 10^{6}$
F7	Mean	$2.059\times 10^{3}$	$2.222\times 10^{3}$	$2.220\times 10^{3}$	$2.238\times 10^{3}$	$2.232\times 10^{3}$	$2.120\times 10^{3}$	$2.167\times 10^{3}$	$2.211\times 10^{3}$	$2.389\times 10^{3}$	$2.080\times 10^{3}$
	Std	$2.482\times 10^{1}$	$3.612\times 10^{1}$	$4.477\times 10^{1}$	$7.665\times 10^{1}$	$8.419\times 10^{1}$	$4.199\times 10^{1}$	$4.819\times 10^{1}$	$4.563\times 10^{1}$	$1.081\times 10^{2}$	$1.189\times 10^{1}$
F8	Mean	$2.224\times 10^{3}$	$2.426\times 10^{3}$	$2.445\times 10^{3}$	$2.284\times 10^{3}$	$2.585\times 10^{3}$	$2.267\times 10^{3}$	$2.307\times 10^{3}$	$2.357\times 10^{3}$	$2.682\times 10^{3}$	$2.239\times 10^{3}$
	Std	$2.591\times 10^{0}$	$1.411\times 10^{2}$	$1.768\times 10^{2}$	$5.336\times 10^{1}$	$2.292\times 10^{2}$	$5.155\times 10^{1}$	$1.006\times 10^{2}$	$1.177\times 10^{2}$	$3.593\times 10^{2}$	$4.837\times 10^{0}$
F9	Mean	$2.483\times 10^{3}$	$3.370\times 10^{3}$	$3.466\times 10^{3}$	$2.562\times 10^{3}$	$3.128\times 10^{3}$	$2.524\times 10^{3}$	$3.106\times 10^{3}$	$2.984\times 10^{3}$	$3.720\times 10^{3}$	$2.481\times 10^{3}$
	Std	$2.797\times 10^{0}$	$2.510\times 10^{2}$	$4.591\times 10^{2}$	$5.032\times 10^{1}$	$2.716\times 10^{2}$	$8.769\times 10^{1}$	$2.643\times 10^{2}$	$2.319\times 10^{2}$	$4.828\times 10^{2}$	$3.003\times 10^{-1}$
F10	Mean	$2.557\times 10^{3}$	$6.406\times 10^{3}$	$6.867\times 10^{3}$	$4.624\times 10^{3}$	$5.569\times 10^{3}$	$4.134\times 10^{3}$	$4.887\times 10^{3}$	$5.906\times 10^{3}$	$6.784\times 10^{3}$	$2.513\times 10^{3}$
	Std	$1.987\times 10^{2}$	$8.191\times 10^{2}$	$5.481\times 10^{2}$	$1.297\times 10^{3}$	$1.072\times 10^{3}$	$9.890\times 10^{2}$	$1.386\times 10^{3}$	$1.256\times 10^{3}$	$1.494\times 10^{3}$	$5.742\times 10^{0}$
F11	Mean	$2.926\times 10^{3}$	$7.819\times 10^{3}$	$8.785\times 10^{3}$	$3.512\times 10^{3}$	$8.641\times 10^{3}$	$4.159\times 10^{3}$	$7.084\times 10^{3}$	$7.964\times 10^{3}$	$9.407\times 10^{3}$	$4.983\times 10^{3}$
	Std	$7.158\times 10^{1}$	$4.618\times 10^{2}$	$5.661\times 10^{2}$	$2.950\times 10^{2}$	$2.040\times 10^{3}$	$1.893\times 10^{3}$	$1.046\times 10^{3}$	$8.226\times 10^{2}$	$6.669\times 10^{2}$	$4.794\times 10^{2}$
F12	Mean	$2.988\times 10^{3}$	$3.473\times 10^{3}$	$3.628\times 10^{3}$	$3.081\times 10^{3}$	$3.855\times 10^{3}$	$3.054\times 10^{3}$	$3.702\times 10^{3}$	$3.511\times 10^{3}$	$3.951\times 10^{3}$	$2.945\times 10^{3}$
	Std	$2.870\times 10^{1}$	$1.578\times 10^{2}$	$2.595\times 10^{2}$	$1.287\times 10^{2}$	$2.568\times 10^{2}$	$7.158\times 10^{1}$	$1.459\times 10^{2}$	$2.696\times 10^{2}$	$3.468\times 10^{2}$	$2.368\times 10^{0}$
Friedman Rank		**1.500**	7.333	8.167	4.667	6.833	2.917	5.000	6.667	9.917	2.000
Final Rank		**1**	8	9	4	7	3	5	6	10	2

**Table 9:** Wilcoxon rank sum test results for CEC2020 in dimension 20.

Fun	ICOA	COA	WOA	AOA	BKA	HOA	NCHHO	ISCSO	LSHADE_cnEpSin
F1	$4.08\times 10^{-11}$	$3.02\times 10^{-11}$	$2.57\times 10^{-7}$	$2.87\times 10^{-10}$	$2.60\times 10^{-8}$	$1.29\times 10^{-9}$	$3.02\times 10^{-11}$	$3.02\times 10^{-11}$	$1.16\times 10^{-7}$
F2	$3.02\times 10^{-11}$	$3.02\times 10^{-11}$	$8.10\times 10^{-10}$	$3.02\times 10^{-11}$	$9.07\times 10^{-3}$	$3.02\times 10^{-11}$	$3.02\times 10^{-11}$	$3.02\times 10^{-11}$	$5.40\times 10^{-1}$
F3	$3.02\times 10^{-11}$	$3.02\times 10^{-11}$	$3.02\times 10^{-11}$	$3.02\times 10^{-11}$	$3.02\times 10^{-11}$	$3.02\times 10^{-11}$	$3.02\times 10^{-11}$	$3.02\times 10^{-11}$	$7.22\times 10^{-6}$
F4	$3.02\times 10^{-11}$	$3.02\times 10^{-11}$	$4.62\times 10^{-10}$	$3.02\times 10^{-11}$	$1.62\times 10^{-1}$	$7.39\times 10^{-11}$	$3.02\times 10^{-11}$	$3.02\times 10^{-11}$	$4.98\times 10^{-11}$
F5	$3.02\times 10^{-11}$	$3.02\times 10^{-11}$	$8.99\times 10^{-11}$	$5.07\times 10^{-10}$	$9.03\times 10^{-4}$	$7.09\times 10^{-8}$	$3.02\times 10^{-11}$	$3.02\times 10^{-11}$	$3.64\times 10^{-2}$
F6	$3.02\times 10^{-11}$	$3.02\times 10^{-11}$	$8.99\times 10^{-11}$	$3.02\times 10^{-11}$	$2.05\times 10^{-3}$	$3.02\times 10^{-11}$	$3.02\times 10^{-11}$	$3.02\times 10^{-11}$	$3.02\times 10^{-11}$
F7	$3.02\times 10^{-11}$	$3.02\times 10^{-11}$	$3.02\times 10^{-11}$	$3.34\times 10^{-11}$	$7.09\times 10^{-8}$	$6.70\times 10^{-11}$	$3.34\times 10^{-11}$	$3.02\times 10^{-11}$	$1.17\times 10^{-4}$
F8	$3.02\times 10^{-11}$	$3.02\times 10^{-11}$	$3.02\times 10^{-11}$	$3.02\times 10^{-11}$	$2.87\times 10^{-10}$	$3.69\times 10^{-11}$	$3.02\times 10^{-11}$	$3.02\times 10^{-11}$	$3.02\times 10^{-11}$
F9	$3.02\times 10^{-11}$	$3.02\times 10^{-11}$	$3.02\times 10^{-11}$	$3.02\times 10^{-11}$	$1.49\times 10^{-4}$	$3.02\times 10^{-11}$	$3.02\times 10^{-11}$	$3.02\times 10^{-11}$	$5.11\times 10^{-1}$
F10	$3.02\times 10^{-11}$	$3.02\times 10^{-11}$	$2.02\times 10^{-8}$	$4.50\times 10^{-11}$	$1.69\times 10^{-9}$	$4.20\times 10^{-10}$	$6.07\times 10^{-11}$	$5.49\times 10^{-11}$	$9.51\times 10^{-6}$
F11	$3.02\times 10^{-11}$	$3.02\times 10^{-11}$	$4.08\times 10^{-11}$	$3.02\times 10^{-11}$	$7.12\times 10^{-9}$	$3.02\times 10^{-11}$	$3.02\times 10^{-11}$	$3.02\times 10^{-11}$	$3.02\times 10^{-11}$
F12	$3.02\times 10^{-11}$	$3.02\times 10^{-11}$	$8.88\times 10^{-6}$	$3.02\times 10^{-11}$	$1.75\times 10^{-5}$	$3.02\times 10^{-11}$	$3.02\times 10^{-11}$	$3.02\times 10^{-11}$	$4.08\times 10^{-11}$
+/−/=	12/0/0	12/0/0	12/0/0	12/0/0	10/1/1	12/0/0	12/0/0	12/0/0	7/2/3

**Table 10:** Friedman test results for all algorithms on different test sets.

Index	MSECOA	ICOA	COA	WOA	AOA	BKA	HOA	NCHHO	ISCSO	LSHADE_cnEpSin	Function Sets/Dime
Friedman Rank	**1.207**	6.793	8.000	4.897	6.862	2.897	5.655	6.724	9.966	2.000	CEE2017-10
Final Rank	**1**	7	9	4	8	3	5	6	10	2	CEE2017-10
Friedman Rank	**1.103**	7.552	8.414	4.379	6.690	2.759	4.897	6.586	10.000	2.621	CEE2017-30
Final Rank	**1**	8	9	4	7	3	5	6	10	2	CEE2017-30
Friedman Rank	**1.500**	7.333	8.167	4.667	6.833	2.917	5.000	6.667	9.917	2.000	CEE2022-20
Final Rank	**1**	8	9	4	7	3	5	6	10	2	CEE2022-20
Sum Rank	**3**	23	27	12	22	9	15	18	30	6	
Total Rank	**1**	8	9	4	7	3	5	6	10	2	

**Table 11:** Results of the welded beam structural design problem.

Algorithm	$x_{1}$	$x_{2}$	Best	Mean	Std	Rank
MSECOA	0.7887	0.4082	**263.8958**	**263.8958**	$2.98556\times 10^{-14}$	**1**
ICOA	0.7898	0.4051	263.8988	263.9568	0.0563	5
COA	0.7879	0.4105	263.8971	263.9549	0.0529	4
WOA	0.7890	0.4072	263.8959	264.8607	1.4424	8
AOA	0.7946	0.3924	263.9812	265.3566	3.3525	9
BKA	0.7887	0.4082	**263.8958**	263.8963	0.0007	3
HOA	0.7891	0.4071	263.8960	263.9713	0.0879	6
NCHHO	0.7891	0.4070	263.8960	264.4827	0.8804	7
ISCSO	0.7965	0.3867	263.9454	266.8552	2.8259	10
LSHADE_cnEpSin	0.7887	0.4082	**263.8958**	**263.8958**	$1.46046\times 10^{-05}$	2

**Table 12:** Results of welded beam structural design problem.

Algorithm	*h*	*l*	*t*	*b*	Best	Mean	Std	Rank
MSECOA	0.2057	3.2349	9.0366	0.2057	**1.6928**	**1.6928**	**0.0001**	**1**
ICOA	0.1862	3.9999	8.9225	0.2300	1.9303	2.7179	0.4480	8
COA	0.2104	3.7201	8.6192	0.2262	1.8437	2.3816	0.2422	6
WOA	0.1913	3.6258	9.0340	0.2058	1.7236	2.4537	0.7443	7
AOA	0.1965	3.0970	10.0000	0.2047	1.8156	2.2361	0.2753	4
BKA	0.2057	3.2353	9.0358	0.2058	1.6930	1.8931	0.5129	3
HOA	0.2934	2.6388	7.3351	0.3235	2.1507	3.1038	0.4459	9
NCHHO	0.2013	3.3533	9.0795	0.2055	1.7080	2.3449	0.7374	5
ISCSO	0.1979	4.2002	9.6899	0.2044	1.9157	$9.1641\times 10^{99}$	$3.1053\times 10^{100}$	10
LSHADE_cnEpSin	0.2057	3.2361	9.0365	0.2057	1.6930	1.7112	0.0302	2

**Table 13:** Results of tension/compression spring design problem.

Algorithm	*d*	*D*	*N*	Best	Mean	Std	Rank
MSECOA	0.0517	0.3567	11.2892	**0.012665**	**0.012665**	$7.8035\times 10^{-8}$	**1**
ICOA	0.0500	0.3161	14.3177	0.012894	$1.6579\times 10^{99}$	$4.9779\times 10^{99}$	9
COA	0.0516	0.3544	11.4419	0.012683	$7.6036\times 10^{98}$	$4.1647\times 10^{99}$	7
WOA	0.0513	0.3470	11.8798	0.012668	0.013502	$6.9757\times 10^{-4}$	3
AOA	0.0500	0.3104	15.0000	0.013193	0.013846	$3.1890\times 10^{-3}$	4
BKA	0.0518	0.3595	11.1294	**0.012665**	$7.6036\times 10^{98}$	$4.1647\times 10^{99}$	7
HOA	0.0516	0.3549	11.3974	0.012668	0.014367	$1.2166\times 10^{-3}$	6
NCHHO	0.0539	0.4126	8.6363	0.012757	0.014119	$1.1151\times 10^{-3}$	5
ISCSO	0.0661	0.8050	4.2486	0.021963	$6.0657\times 10^{99}$	$1.4491\times 10^{100}$	10
LSHADE_cnEpSin	0.0513	0.3482	11.8090	0.012668	0.012699	$3.2278\times 10^{-5}$	2

**Table 14:** Results of pressure vessel design problem.

Algorithm	$T_{s}$	$T_{s}$	*R*	*L*	Best	Mean	Std	Rank
MSECOA	0.7782	0.3846	40.3196	200.0000	**5885.3328**	**5885.4843**	**0.6666**	**1**
ICOA	1.2409	2.6352	61.6431	76.7312	23,715.1692	87,460.1428	45,702.0842	9
COA	1.3691	1.1215	56.0524	56.5861	11,388.2108	73,119.0929	52,805.4449	8
WOA	0.8175	0.4224	41.4994	184.2044	6123.4849	9566.4849	4663.8196	3
AOA	1.1984	0.5752	54.1501	136.2680	10,665.3792	69,181.7606	77,481.3905	7
BKA	0.7783	0.3847	40.3263	199.9070	5885.5533	13,798.6379	34,303.5253	5
HOA	0.8813	0.6617	44.0702	158.7985	7193.0785	10,177.4336	4496.0008	4
NCHHO	0.9757	0.4467	46.7089	126.8062	6594.3919	24,205.6310	27,288.7444	6
ISCSO	2.7814	2.8772	70.5541	124.9528	54,618.2573	213,250.5202	101,961.8458	10
LSHADE_cnEpSin	0.7796	0.3859	40.3938	198.9785	5889.7343	6057.2659	218.7573	2

**Table 15:** Degree reduction error results for example 1.

Algorithm	Coordinates	Mean (ε)	Std (ε)	Min (ε)
COA	$q_{0}=\left(0.1,0\right),q_{1}=\left(-0.0605,0.1587\right),q_{2}=\left(0.2652,0.3195\right),$	$2.180\times 10^{-3}$	$2.022\times 10^{-3}$	$3.900\times 10^{-5}$
	$q_{3}=\left(0.6998,0.1790\right),q_{4}=\left(0.5,0\right)$			
MSECOA	$q_{0}=\left(0.1,0\right),q_{1}=\left(-0.0774,0.1742\right),q_{2}=\left(0.3006,0.3142\right),$	$3.671\times 10^{-5}$	$2.710\times 10^{-6}$	$3.351\times 10^{-5}$
	$q_{3}=\left(0.6769,0.1738\right),q_{4}=\left(0.5,0\right)$			
ICOA	$q_{0}=\left(0.1,0\right),q_{1}=\left(-0.0937,0.2500\right),q_{2}=\left(0.3804,0.2500\right),$	$3.149\times 10^{-3}$	$2.029\times 10^{-3}$	$6.557\times 10^{-4}$
	$q_{3}=\left(0.6000,0.2500\right),q_{4}=\left(0.5,0\right)$			
WOA	$q_{0}=\left(0.1,0\right),q_{1}=\left(-0.0396,0.1594\right),q_{2}=\left(0.2076,0.3759\right),$	$1.786\times 10^{-3}$	$1.977\times 10^{-3}$	$1.533\times 10^{-4}$
	$q_{3}=\left(0.7621,0.1255\right),q_{4}=\left(0.5,0\right)$			
HOA	$q_{0}=\left(0.1,0\right),q_{1}=\left(-0.0869,0.1825\right),q_{2}=\left(0.3115,0.3159\right),$	$5.121\times 10^{-4}$	$5.019\times 10^{-4}$	$5.443\times 10^{-5}$
	$q_{3}=\left(0.6773,0.1744\right),q_{4}=\left(0.5,0\right)$			

**Table 16:** Degree reduction error results for example 2.

Algorithm	Coordinates	Mean (ε)	Std (ε)	Min (ε)
COA	$q_{0}=\left(-0.871,0.408\right),q_{1}=\left(-0.6119,0.8805\right),q_{2}=\left(0.2604,-0.0209\right),$	$1.295\times 10^{-2}$	$4.820\times 10^{-3}$	$4.606\times 10^{-3}$
	$q_{3}=\left(0.3916,1.1019\right),q_{4}=\left(0.871,0.409\right)$			
MSECOA	$q_{0}=\left(-0.871,0.408\right),q_{1}=\left(-0.6290,1.1316\right),q_{2}=\left(0.0017,-0.3108\right),$	$5.704\times 10^{-4}$	$4.420\times 10^{-6}$	$5.628\times 10^{-4}$
	$q_{3}=\left(0.6280,1.1331\right),q_{4}=\left(0.871,0.409\right)$			
ICOA	$q_{0}=\left(-0.871,0.408\right),q_{1}=\left(-0.6959,1.1275\right),q_{2}=\left(0.1221,-0.3068\right),$	$4.988\times 10^{-3}$	$1.755\times 10^{-3}$	$7.190\times 10^{-4}$
	$q_{3}=\left(0.5288,1.1379\right),q_{4}=\left(0.871,0.409\right)$			
WOA	$q_{0}=\left(-0.871,0.408\right),q_{1}=\left(-0.4961,1.0977\right),q_{2}=\left(-0.2040,-0.3108\right),$	$2.325\times 10^{-3}$	$1.096\times 10^{-3}$	$9.846\times 10^{-4}$
	$q_{3}=\left(0.6934,1.1771\right),q_{4}=\left(0.871,0.409\right)$			
HOA	$q_{0}=\left(-0.871,0.408\right),q_{1}=\left(-0.6451,0.8733\right),q_{2}=\left(0.1713,0.0296\right),$	$7.412\times 10^{-3}$	$2.819\times 10^{-3}$	$3.153\times 10^{-3}$
	$q_{3}=\left(0.5055,1.0026\right),q_{4}=\left(0.871,0.409\right)$			

**Table 17:** Degree reduction error results for example 3.

Algorithm	Coordinates	Mean (ε)	Std (ε)	Min (ε)
COA	$q_{0}=\left(-0.5,0\right),q_{1}=\left(-0.7894,0.7768\right),q_{2}=\left(0.0600,1.1144\right),$	$3.368\times 10^{-2}$	$2.899\times 10^{-2}$	$4.386\times 10^{-3}$
	$q_{3}=\left(2.6733,0.9246\right),q_{4}=\left(1.7,0\right)$			
MSECOA	$q_{0}=\left(-0.5,0\right),q_{1}=\left(-1.1377,0.6125\right),q_{2}=\left(0.5999,1.6111\right),$	$1.059\times 10^{-5}$	$1.070\times 10^{-5}$	$6.720\times 10^{-6}$
	$q_{3}=\left(2.3374,0.6119\right),q_{4}=\left(1.7,0\right)$			
ICOA	$q_{0}=\left(-0.5,0\right),q_{1}=\left(-1.0757,0.6892\right),q_{2}=\left(0.6904,1.5395\right),$	$4.699\times 10^{-3}$	$2.565\times 10^{-3}$	$1.075\times 10^{-3}$
	$q_{3}=\left(2.2891,0.5899\right),q_{4}=\left(1.7,0\right)$			
WOA	$q_{0}=\left(-0.5,0\right),q_{1}=\left(-1.0499,0.6985\right),q_{2}=\left(0.5199,1.5090\right),$	$3.980\times 10^{-3}$	$2.659\times 10^{-3}$	$3.192\times 10^{-4}$
	$q_{3}=\left(2.3523,0.6317\right),q_{4}=\left(1.7,0\right)$			
HOA	$q_{0}=\left(-0.5,0\right),q_{1}=\left(-1.2617,0.8366\right),q_{2}=\left(0.8275,1.1947\right),$	$5.643\times 10^{-3}$	$3.959\times 10^{-3}$	$1.577\times 10^{-3}$
	$q_{3}=\left(2.2081,0.8827\right),q_{4}=\left(1.7,0\right)$			

**Table 18:** Degree reduction error results for example 4.

Algorithm	Coordinates	Mean (ε)	Std (ε)	Min (ε)
COA	$q_{0}=\left(-0.3,0\right),q_{1}=\left(-1.5113,1.1532\right),q_{2}=\left(2.6916,1.1674\right),$	$1.844\times 10^{-2}$	$1.867\times 10^{-2}$	$1.650\times 10^{-3}$
	$q_{3}=\left(1.5,0\right)$			
MSECOA	$q_{0}=\left(-0.3,0\right),q_{1}=\left(-1.5695,1.1602\right),q_{2}=\left(2.7704,1.1545\right),$	$1.353\times 10^{-3}$	$8.900\times 10^{-7}$	$1.350\times 10^{-3}$
	$q_{3}=\left(1.5,0\right)$			
ICOA	$q_{0}=\left(-0.3,0\right),q_{1}=\left(-1.5965,1.1989\right),q_{2}=\left(2.7993,1.1578\right),$	$2.567\times 10^{-3}$	$9.558\times 10^{-4}$	$2.518\times 10^{-3}$
	$q_{3}=\left(1.5,0\right)$			
WOA	$q_{0}=\left(-0.3,0\right),q_{1}=\left(-1.5844,1.1599\right),q_{2}=\left(2.7882,1.1457\right),$	$1.746\times 10^{-3}$	$3.518\times 10^{-4}$	$1.354\times 10^{-3}$
	$q_{3}=\left(1.5,0\right)$			
HOA	$q_{0}=\left(-0.3,0\right),q_{1}=\left(-1.5632,1.1817\right),q_{2}=\left(2.7636,1.1395\right),$	$6.389\times 10^{-3}$	$7.880\times 10^{-3}$	$1.382\times 10^{-3}$
	$q_{3}=\left(1.5,0\right)$			

## Data Availability

All data generated or analyzed during the study are included in this published article.
